# Recent advancements in single-cell metabolic analysis for pharmacological research

**DOI:** 10.1016/j.jpha.2023.08.014

**Published:** 2023-08-23

**Authors:** Ying Hou, Hongren Yao, Jin-Ming Lin

**Affiliations:** Beijing Key Laboratory of Microanalytical Methods and Instrumentation, Key Laboratory of Bioorganic Phosphorus Chemistry & Chemical Biology (Ministry of Education), Department of Chemistry, Tsinghua University, Beijing, 100084, China

**Keywords:** Single-cell, Metabolism, Pharmacology, Drug response, Microfluidics

## Abstract

Cellular heterogeneity is crucial for understanding tissue biology and disease pathophysiology. Pharmacological research is being advanced by single-cell metabolic analysis, which offers a technique to identify variations in RNA, proteins, metabolites, and drug molecules in cells. In this review, the recent advancement of single-cell metabolic analysis techniques and their applications in drug metabolism and drug response are summarized. High-precision and controlled single-cell isolation and manipulation are provided by microfluidics-based methods, such as droplet microfluidics, microchamber, open microfluidic probe, and digital microfluidics. They are used in tandem with variety of detection techniques, including optical imaging, Raman spectroscopy, electrochemical detection, RNA sequencing, and mass spectrometry, to evaluate single-cell metabolic changes in response to drug administration. The advantages and disadvantages of different techniques are discussed along with the challenges and future directions for single-cell analysis. These techniques are employed in pharmaceutical analysis for studying drug response and resistance pathway, therapeutic targets discovery, and in vitro disease model evaluation.

## Highlights

• Single-cell metabolic analysis for pharmacological research is reviewed.
• Microfluidic methods are vital for precise and controlled single-cell isolation and manipulation.
• Technologies, such as optical imaging and mass spectrometry, assess the heterogeneity of drug-induced metabolic changes.
• Single-cell metabolic analysis aids in studying drug pathways, new therapeutic targets, and disease models.

## Graphical abstract

**Figure:**
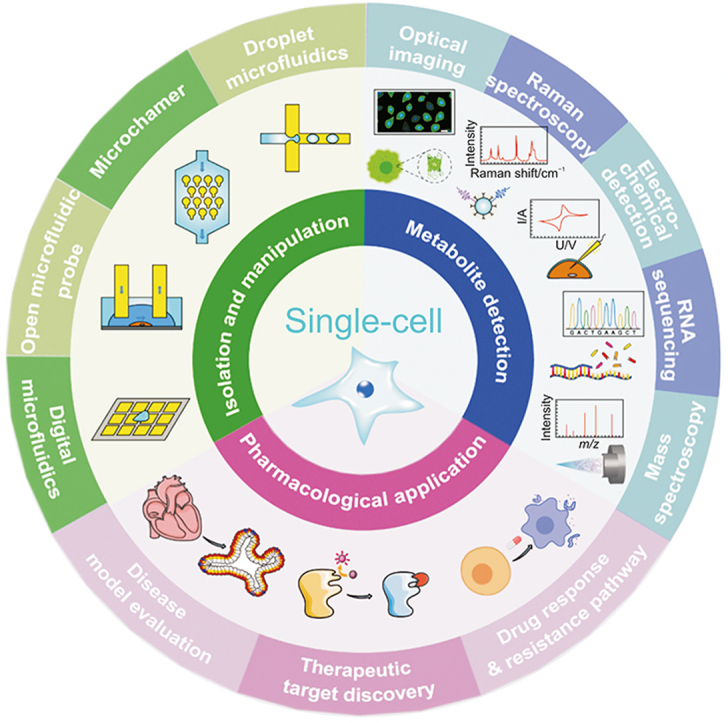

## 1 Introduction

Cellular heterogeneity, which refers to the phenotypic variation among individual cells within the same source or culture environment, is widespread and plays a pivotal role in embryonic development, cell differentiation, and disease progression [1]. Traditional cell research uses the average measurement value of cell populations to represent cellular and tissue behavior, potentially obscuring information regarding small cell subtypes and even deviating from the behavior of the majority of cells [2]. Moreover, accumulating studies have demonstrated that the behavior of a select few cells may hold greater importance within specific physiological environments [3]. The single-cell analysis method makes it possible to gather information on the morphology, gene expression, metabolites, and other properties of individual cells. Cell typing and behavior evaluation are subsequently carried out using multi-dimensional combined statistical analysis. Utilizing single-cell analysis techniques in drug response and therapy research can offer a molecular-scale understanding of drug-cell interactions, thus advancing our knowledge of intracellular pharmacokinetics and pharmacology [4].

In recent years, many studies have demonstrated the importance of dynamic cellular metabolism and behavior under drug administration for assessing drug efficacy and understanding drug mechanisms [5,6]. However, the requirement to examine single-cell metabolites presents multiple challenges for conventional analytical methods. One of the prerequisites is a gentle and highly accurate manipulation technology. Operating fluids in micrometer and nanometer scales, microfluidics enables controlled environmental stimulation and precise cell separation [7,8]. They also have high throughput and easy integration with detection units [9,10]. Additionally, cell metabolites have characteristics including trace quantities, limited amplification potential, high dynamics, and a wide concentration range. As a result, rapid and highly sensitive detection technologies are required. Furthermore, detection techniques with comprehensive molecular detection capabilities are required due to the diversity and structural complexity of cellular metabolites. Multiple single-cell analysis methods, including optical imaging [11], Raman spectroscopy [12], electrochemical methods [13], single-cell RNA sequencing (scRNA-seq) [14], and mass spectrometry (MS) [15] are recently developed. For pharmaceutical analysis, these methods allow for the tracking of drug molecule uptake, distribution, and structural alterations within cells as well as the evaluation of how pharmaceuticals affect cell activity and metabolism. These findings are also utilized to identify unique cells and their physiological importance, to reveal cellular interactions in the tumor microenvironment, and to provide new therapeutic options. As a result, they offer invaluable insights into the study of diseases and clinical medicine [16].

**Fig. 1:**
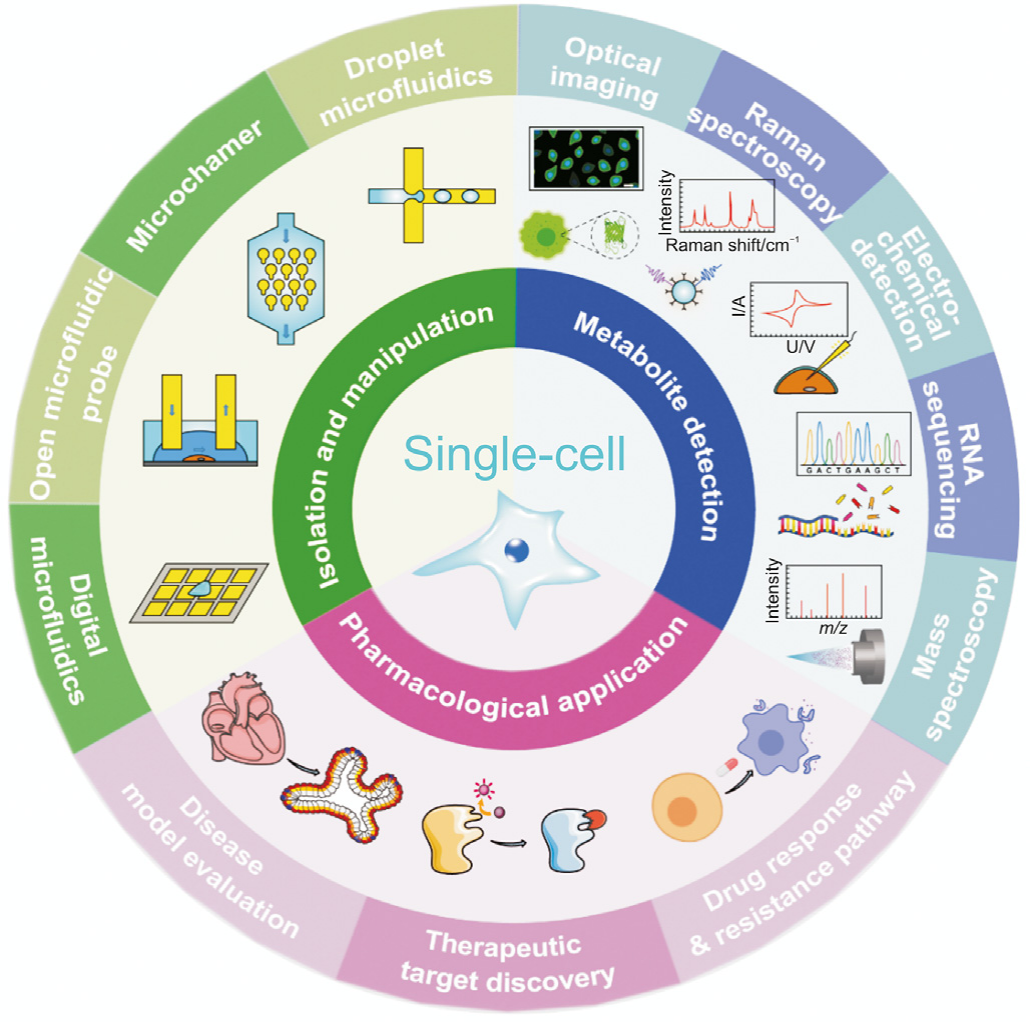
Summary of the single-cell isolation, manipulation, metabolic detection technologies and their pharmacological applications.

In this review, we focus on the most current advancements in single-cell analysis methods for pharmaceutical analysis. The common single-cell manipulation and detection techniques are covered, highlighting their respective advantages and limitations. Then a summary of the applications of single-cell analysis technology in drug metabolism and response, including studies on drug response and resistance, the discovery of novel therapeutic targets, and the evaluation of the drug delivery model are provided (Fig. 1). Finally, we discuss the future directions and main challenges for this field.

## 2 Technologies for single-cell metabolic analysis

### 2.1 The single-cell isolation and manipulation methods

Single-cell analysis starts with the separation and adaptable manipulation of individual cells. Microfluidics enables the separation, sorting, and sampling of single cells through the near-single-cell scale control of fluids. Recent single-cell isolation and manipulation techniques include droplet microfluidics, microchamber, open microfluidic probe, digital microfluidics, etc. Table 1 evaluates the three important factors for using these technologies for single-cell analysis: throughput, operability, and compatibility with detection technologies.

**Table 1:** Single-cell manipulation methods.

Method	Throughput	Operability	Compatibility with detection technologies
Droplet microfluidics	High	Medium	High
Microchamber	Medium	Low	Low
Open microfluidic probe	Low	High	Medium
Digital microfluidics	Low	High	High

#### 2.1.1 Droplet microfluidics

Droplet microfluidics offers an efficient method of separating single cells by encapsulating them within picoliter (pL) and nanoliter (nL) droplets. Microfluidic chip design enables a variety of droplet manipulations, including fusion, division, mixing, storage, and sorting [17]. As a result, cells were stimulated, lysed, and sample processed. Droplet microfluidics also provides the benefits of high throughput, minimal reagent consumption, increased sensitivity, and facile compatibility with subsequent detection methods. As a widely employed method for manipulating cells, droplet microfluidics serves as the foundation for most high-throughput single-cell sequencing technologies. The approaches of droplet production and manipulation have been extensively reviewed [18,19]. Here we focus on the utilization of droplet microfluidics as a pre-treatment technique and its integration with detection technologies.

**Fig. 2:**
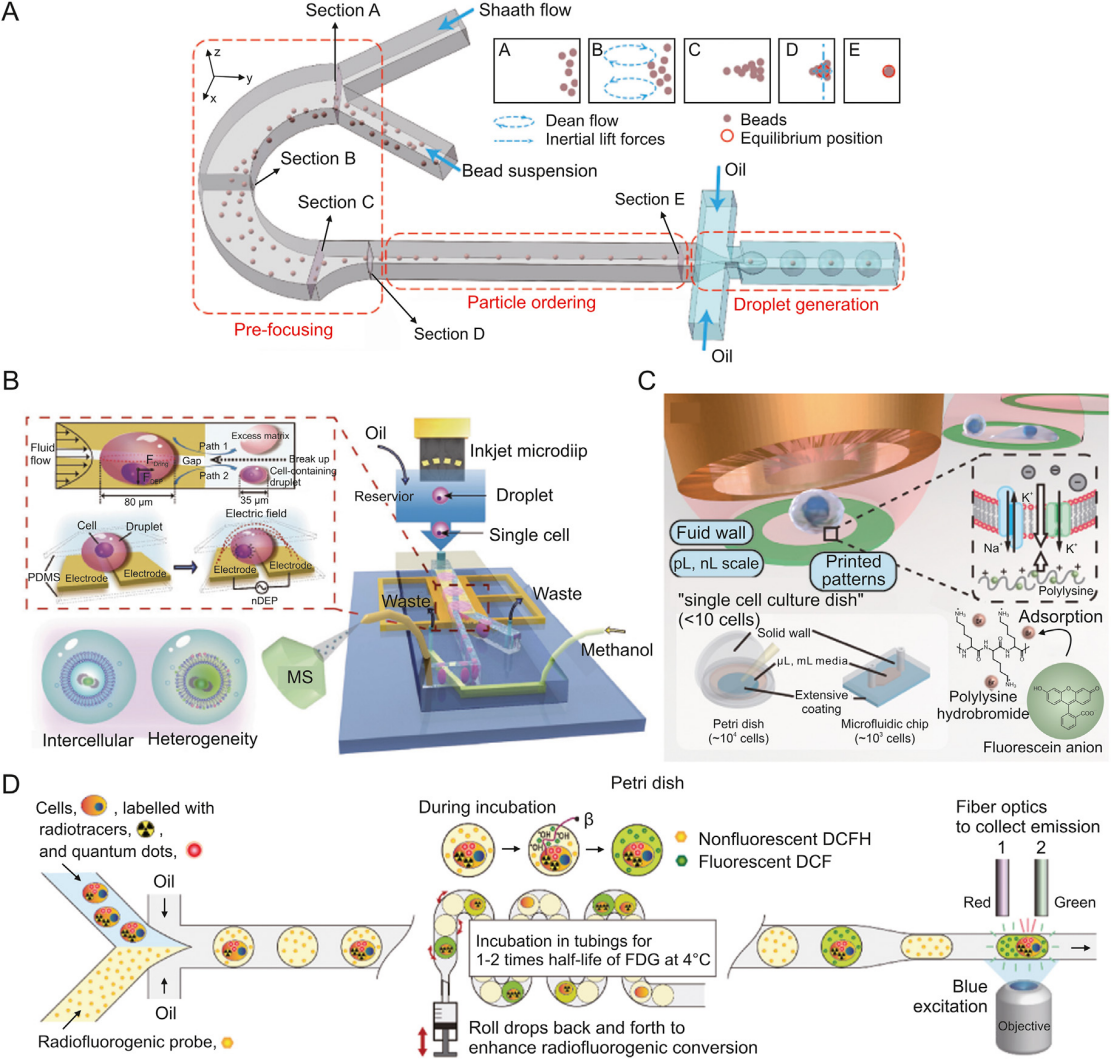
Droplet microfluidics-based single-cell isolation and manipulation methods. (A) Schematic diagram of the fast bead arrangement and droplet generation system by creating the sheath flow, Dean vortex, and compression flow sequentially in the channel. Reprinted with permission from Ref. [20], copyright 2022 Elsevier. (B) Schematic diagram of the integrated droplet microfluidic platform for online single-cell encapsulation, pre-processing and lipid profiling. Reprinted with permission from Ref. [22], copyright 2020 Wiley-VCH GmbH. (C) Schematic diagram of the open droplet microfluidic platform for culturing adherent single cells. Reprinted with permission from Ref. [25], copyright 2022 Wiley-VCH GmbH. (D) Schematic diagram of the processes of the flow radiocytometry, including droplet generation, incubation, and fluorescence detection. Reprinted with permission from Ref. [27], copyright 2021 Elsevier.

The problem of the single-cell encapsulation rate has long been a barrier to the use of droplet microfluidic technology in single-cell analysis. The problem of the single-cell encapsulation rate has long been a barrier to the use of droplet microfluidic technology in single-cell analysis. Due to the Poisson distribution, high cell concentrations produce multi-cell droplets that might cause detection errors, while low cell concentrations result in extremely low single-cell encapsulation rates that lower detection throughput and raise analysis costs. To address this problem, Yue et al. [20] developed a compact droplet microfluidic system that achieves ordered particle arrangement and encapsulation within a 1 cm microchannel (Fig. 2A). By combining sheath flow, Dean vortex, and unilateral constrictive structures, they successfully implemented pre-focusing, particle ordering, and droplet generation, achieving an impressive 86% encapsulation rate for single magnetic beads. Additionally, Zhong et al. [21] reported the SELECTS system, an unlabeled droplet sorting method that effectively removes multi-cells and empty droplets. This microfluidic chip employed two pairs of microelectrodes to detect impedance signals from droplets, accurately determining the cell quantity within each droplet. By selectively activating piezoelectric actuators for droplet manipulation and sorting, this method enriched single-cell droplets to over 90%, irrespective of the initial cell loading density.

Droplet microfluidics are used to analyze the metabolism and behavior of single cells when combined with detection techniques. Zhang et al. [22] proposed an integrated microfluidic system, based on droplet microfluidics and MS, for single-cell phospholipid analysis (Fig. 2B). The process involved inkjet printing in creating droplets, followed by splitting them into daughter droplets with one-fifth of the volume using a dielectrophoresis channel and an asymmetrically bifurcate structure. This reduced interference from the excess culture medium. For online oil removal and sample preparation for direct MS measurement, a demulsification interface with a two-layer channel was employed. Normal astrocyte cells and glioblastoma cells were classified, and the impact of lipopolysaccharide (LPS) stimulation on phospholipid levels in Raw 264.7 macrophage cells was evaluated using this system. Furthermore, the authors introduced an open-space platform with fluid walls for real-time monitoring and in situ MS analysis of tumor cells [23]. They cultivated single-cell droplet arrays on indium tin oxide (ITO) glass, using SNARF-5F to detect lactate secretion and identify potential circulating tumor cells (CTCs). In-situ matrix-assisted laser desorption/ionization mass spectrometry (MALDI-MS) analysis was utilized to evaluate the proportion of phosphatidylcholine (PC) in cells. Wu et al. [24] developed a droplet-based, real-time analysis method to monitor single-cell matrix metalloproteinases (MMP) activity by tracking protein hydrolysis within the droplets. The droplet array was solidified using a thermosetting oil. Fluorescent signals were used to measure the cleavage activity of MMP released by cells, employing a fluorescence resonance energy transfer (FRET) substrate co-cultured with the cells.

Understanding single-cell behavior is crucial to elucidate cellular heterogeneity. Xie et al. [25] introduced an open microfluidic system for culturing adherent single cells (Fig. 2C). Unlike traditional single-cell analysis, this approach created a separate cell culture microenvironment using an inkjet printing-based adhesive substrate patterning method. Cell polarization pattern in the “single cell culture dishes” revealed the potential impact of intercellular communication in single cell assay in conventional culture dishes. Wong et al. [26] presented a precise microscale gel deposition method to investigate the influence of the local matrix environment on single cells. Alginate gel conjugated with the Arg-Gly-Asp (RGD) ligand was crosslinked on the surface of cells coated by CaCO_3_ nanoparticles. The amount of hydrogel deposition was found to be independent of the material composition and elasticity. The study showed that the osteogenic differentiation of mesenchymal stem cells was impacted by the varied membrane tensions caused by variable gel thickness.

Single-cell screening for further investigation is also achieved with droplet microfluidics. Ha et al. [27] developed a flow radiocytometry (FRCM) method to measure radiotracer uptake in single cells (Fig. 2D). Radiolabeled single cells and fluorogenic radio probes were co-encapsulated within droplets to reduce signal crosstalk between droplets. The dim and discontinuous radioactive signals were converted into fluorescent signals in the microdroplets. Using FRCM, the metabolic flux of glucose in various populations of human breast cancer cells was quantified. Zhou et al. [28] developed an integrated single-cell fluorescence sorting chip utilizing pneumatic microvalves to control droplet storage and on-chip incubation. This device enabled hybridoma cell sorting based on antibody phenotypes, achieving an efficiency of approximately 90%. It laid the foundation for miniaturizing and automating single-cell sorting. Agnihotri et al. [29] reported a single-cell cytotoxicity evaluation and selective release method. Natural killer (NK) cells and tumor cells were individually encapsulated in droplets, and the droplets were captured in microchambers. Fast-killing NK cells were identified through fluorescence staining and real-time observation and then selectively recovered with microvalves. Due to its non-destructive of cells, this method was compatible with single-cell omics analysis.

#### 2.1.2 Microchamber

**Fig. 3:**
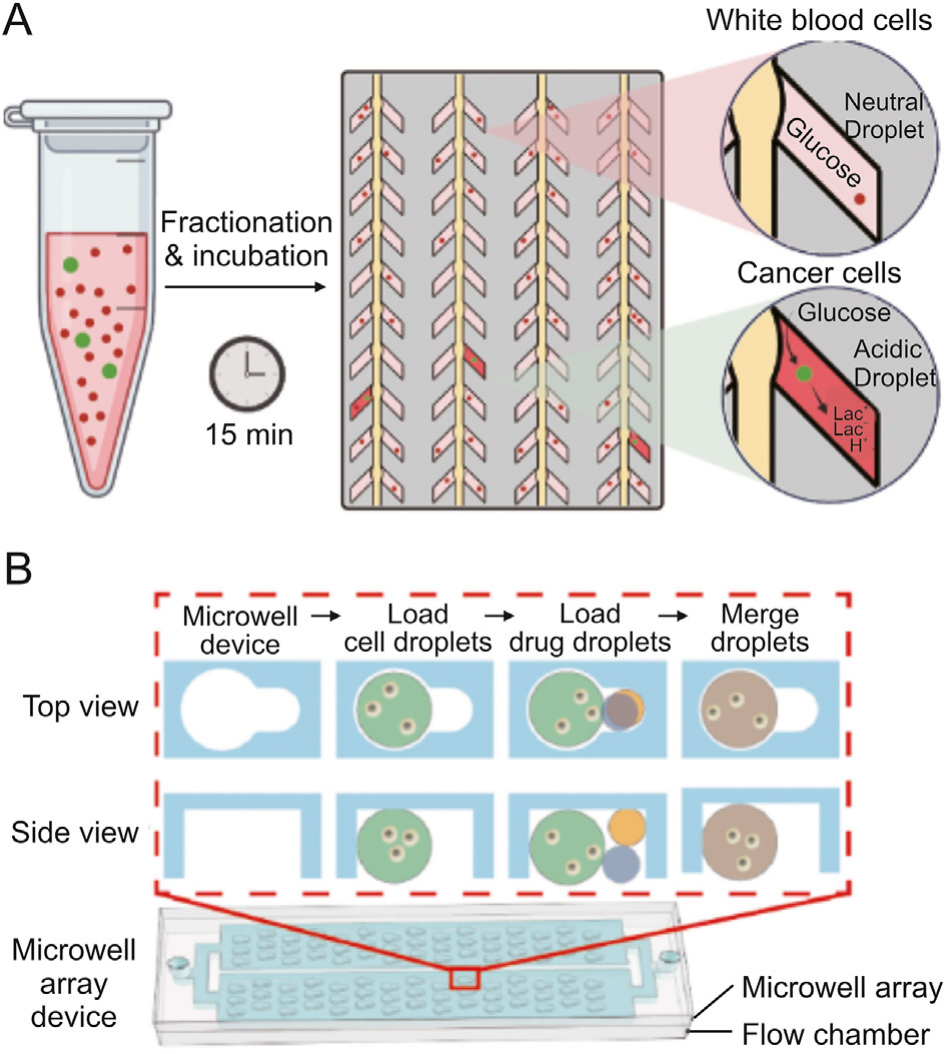
Microchamber-based single-cell isolation and manipulation methods. (A) Schematic diagram of the active CTCs classification microfluidic chip by incubating single cells in the chambers and measuring the lactate production. Reprinted with permission from Ref. [31], copyright 2023 Elsevier. (B) Schematic diagram of the microwell array device for single-cell combinatorial perturbation sequencing. One cell droplet and two drug droplets were randomly loaded in each well. Reprinted with permission from Ref. [32], copyright 2023 Elsevier.

Microchambers are an alternative strategy for single-cell compartmentalization, which enables in situ one-to-one cell-drug stimulation pairing and static real-time observation of cell phenotypes. Khajvand et al. [30] presented a multiplexed single-cell secretome analysis method using integrating single-cell partitioned arrays and antibody barcodes. Static droplets encapsulating single cells were captured in pico-chambers. Microvalves controlled the flow of culture medium to a high-density antibody barcode array in the detection area after incubation, enabling the multiplex identification of cell-secreted proteins. Effector secretion proteins in human differentiated macrophages, human tumor cell lines, and patient samples were evaluated using this method. Radfar et al. [31] developed a high-throughput static droplet microfluidic (SDM) device with 38,400 chambers to isolate and compartmentalize CTCs from blood samples and performed fluorescent detection of their lactate metabolic activity (Fig. 3A). This device could be useful for clinical diagnostics because of its easy operation and independence from pricey detecting tools. Xie et al. [32] reported a combinatorial perturbation sequencing (CP-seq) method using microwell-based droplet random pairing (Fig. 3B). Single-cell droplets were randomly paired with two barcoded drug-containing droplets in the microwell array. Subsequently, the effect of drug combination treatment was evaluated through RNA sequencing. This method was beneficial in high-throughput combinatorial perturbation screening. Zhang et al. [33] proposed a single-cell/barcoded bead coupling method based on the differential flow resistance principle for highly efficient single-cell mRNA sequencing. The chip showed exceptional pairing and cell capture efficiency, maximizing cell use and lowering detection costs. Lin et al. [34] developed a time-resolved microwell single-cell RNA sequencing method called Well-TEMP-seq. In microwells, single cells were dispersed and coupled one-to-one with encoded magnetic beads. 4sU, a biocompatible thymidine analog, was then added to the cells, permitting the differentiation of freshly produced RNAs. The DNA-demethylating drug 5-AZA-CdR's transcriptional kinetics in colorectal cancer cells were discovered using this technique.

#### 2.1.3 Open microfluidic probe

**Fig. 4:**
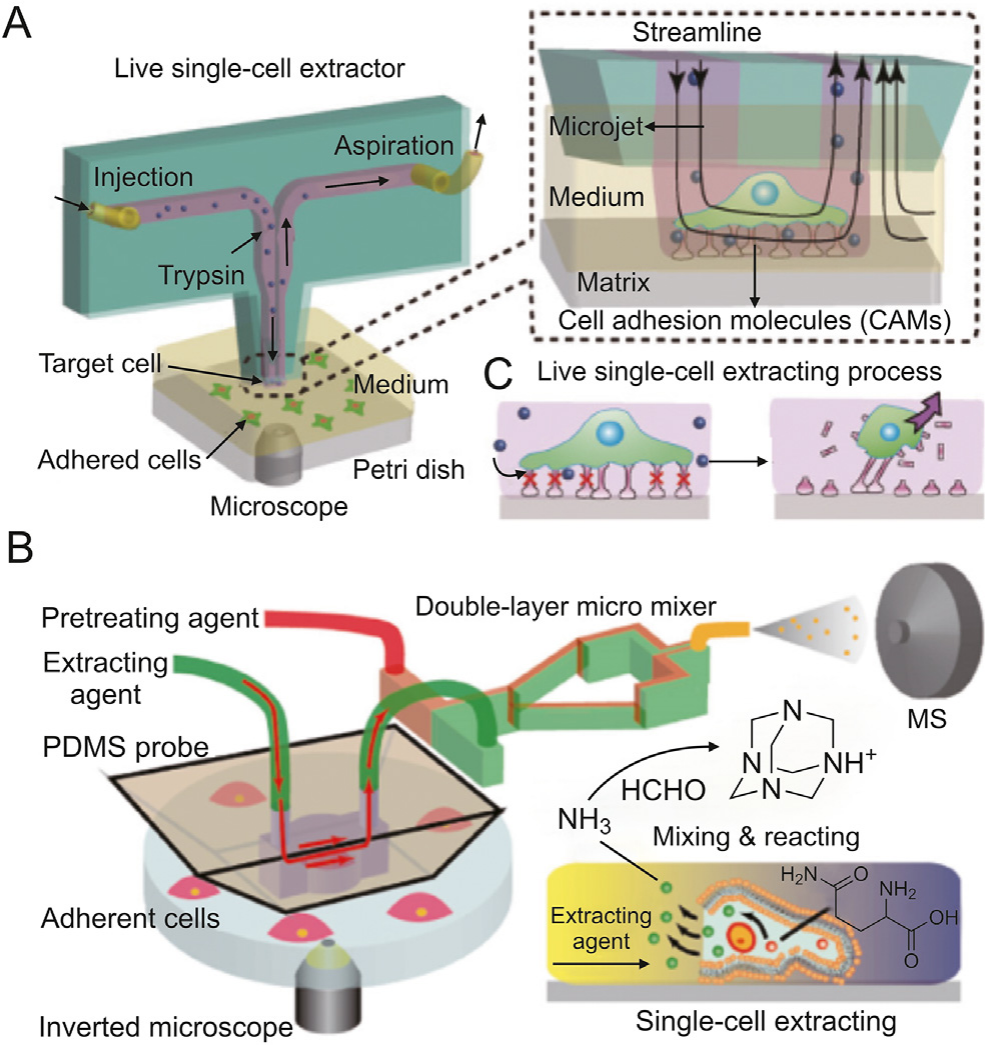
Open microfluidic probe-based single-cell isolation and manipulation methods. (A) Schematic diagram of the open microfluidic probe for live single-cell extraction and adhesion test. Reprinted with permission from Ref. [36], copyright 2018 Wiley-VCH GmbH. (B) Schematic diagram of the adherent single-cell ammonia detection by in-situ extraction, online pretreatment, and MS analysis. Reprinted with permission from Ref. [43], copyright 2023 American Chemical Society.

Open microfluidic probes, which operate on the motion of microscale fluids in the open space below the tips, are well-suited for single-cell manipulation [35]. Lin and co-workers developed an open microfluidic probe for in situ live single-cell extraction and adhesion testing [36]. A stable trypsin cell-extracting solution microjet with a diameter of approximately 100 mm formed under the tip, allowing localized extraction of individual cells (Fig. 4A). The extraction time was corresponded to cell adhesion strength. Correlations were established between adhesion strength and multiple parameters such as cell morphology, intracellular GSH and DHE levels, as well as the state of mitochondria and nuclei. Based on this technology, Lin's lab [[37], [38]] has broadened the research to single-cell extraction on diverse substrates and in response to various environmental stimulation [39]. The fluid-controlled chemical reaction zone can be narrowed down to a few micrometers, allowing for precision localized [40] and subcellular stimulation [41].

Open microfluidic probes also allow for in-situ lysis and sampling of single cells for molecular analysis. A single-cell extraction probe with a temporarily closed extraction chamber was fabricated to prevent the escape of active cellular components during single-cell MS analysis [42]. During the experiment, the probe was tightly sealed onto the target cell, and methanol was introduced to extract both the cell membrane and intracellular phospholipids. The extraction solution was then analyzed using an electrospray quadrupole time-of-flight mass spectrometer (ESI-QTOF-MS). This method effectively detected ten different phosphatidylcholines and achieved the classification of four different tumor cell types. Furthermore, an integrated approach combining single-cell extraction, online derivatization, and MS analysis was developed for the semi-quantitative analysis of trace metabolites [43] (Fig. 4B). An ultralow-volume, high-efficiency micromixer was utilized to convert extracted NH_3_ molecules from single cells into hexamethylenetetramine (N_4_(CH_2_)_6_), which exhibited enhanced MS signals. The results of this study revealed differences in ammonia content among different cell types. Besides, it was shown that cells' ammonia levels were dramatically reduced by hypoxia stimulation.

#### 2.1.4 Digital microfluidics

Digital microfluidics is a novel microfluidic technology that utilizes the principle of electro-wetting to precisely manipulate individual nanoliter droplets. Droplet manipulation, including directional movement, splitting, merging, and mixing, can be precisely managed by adjusting the electrode potential. Digital microfluidic systems are emerging technologies for single-cell multi-omics sample preparation because they can incorporate thermal, magnetic, and optical components.

**Fig. 5:**
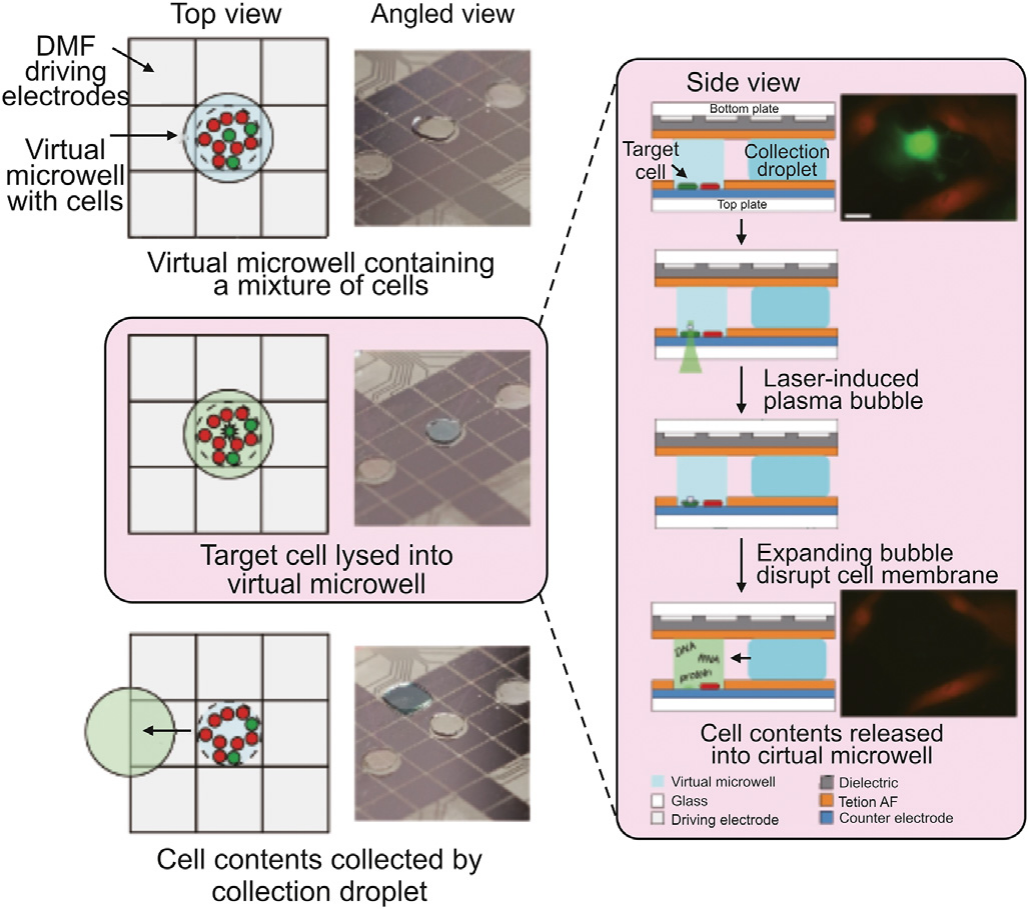
Digital microfluidics (DMF)-based single-cell isolation and manipulation methods. Schematic and physical diagram of the digital microfluidic platform for single-cell isolation and sampling for DNA sequencing, RNA sequencing, and proteomes analysis. Reprinted with permission from Ref. [45], copyright 2020 Springer Nature.

Lammana et al. [44] used digital microfluidics to selectively lysed target single-cell within adherent cell populations for multi-omic analysis. The target cells growing on a digital microfluidic device were selected using artificial intelligence image processing. The targeted cell was then precisely lysed without harming other cells using laser-induced plasma bubbles. Cell lysate droplets were subjected to acquire information on genomic, transcriptomic, and proteomic data using next-generation sequencing, nanoflow liquid chromatography, and MS/MS methods. Zhang et al. [45] established the Cilo-seq digital microfluidic single-cell transcriptome sequencing technology, which represented the first method to build a single-cell whole-transcriptome library on a single chip (Fig. 5). Accurate single-cell transcript profiling was achieved by minimizing nucleic acid loss and exogenous background interference using this integrated platform. Xu et al. [46] reported a digital microfluidics-based platform for single-cell isolation, lysis, DNA/RNA isolation, and in situ nucleic acid amplification. The combination of genomic and transcriptomic sequencing allows for simultaneous genome and transcriptome analysis of a single cell, facilitating the exploration of the correlation between genomic variations and transcriptomic profiles. They discovered two possibly important genes for a poor prognosis in multiple myeloma using this strategy. Digital microfluidics is highly scalable. Therefore, even though it hasn't yet attained high throughput, it has a lot of potential for single-cell analysis.

### 2.2 Single-cell metabolite detection technology

To carry out metabolite analysis at the single-cell level, a variety of detection techniques are combined with single-cell manipulation methods. Commonly used detection techniques include optical imaging, Raman spectroscopy, electrochemical detection, RNA sequencing, and MS. Table 2 provides an outline of their evaluation in terms of throughput, detection target, and cellular destruction. Throughput and multiplexing capabilities are evaluated for the application potential of the technique. The degree of cellular damage determines whether dynamic information on the time scale can be obtained and whether information networks can be constructed by association with other assay techniques. Labeling of intracellular molecules may have an impact on the activity of biomolecules. Types of detected substances evaluate the technology's ability to cover the metabolome.

**Table 2:** Single-cell metabolite detection technologies.

Method	Throughput	Multiplexing	Cellular damage	Intracellular labeling requirement	Types of detected substances	Limitations
Optical imaging	High	Medium	Low	Yes	Molecules can be labeled with fluorescent probes.	Background noise, spectral overlap
Raman Spectroscopy	Medium	Medium	Low	No	Protein, lipid, nucleic acid	Limited in accurate quantification
Electrochemical detection	Low	Low	Medium	No	Molecules with electrochemical activity	Limited analyte coverage
RNA sequencing	High	High	High	No	RNA	Signal dropouts, batch effects
Mass spectrometry	High	High	High	No	Protein, lipid, small molecules etc.	Limited temporal and spatial resolution

#### 2.2.1 Optical imaging

Optical imaging utilize specific probes to label intracellular or extracellular metabolites, providing molecular-level qualitative and quantitative information. High sensitivity, high throughput, and great spatial and temporal resolution are the benefits of optical imaging. Moreover, owing to their non-invasiveness, they enable continuous monitoring of molecular dynamics in live cells and tissues. However, these methods can only detect one or a small number of metabolites at once due to the intricacy of the probe design and the detection resolution of fluorescence channels.

**Fig. 6:**
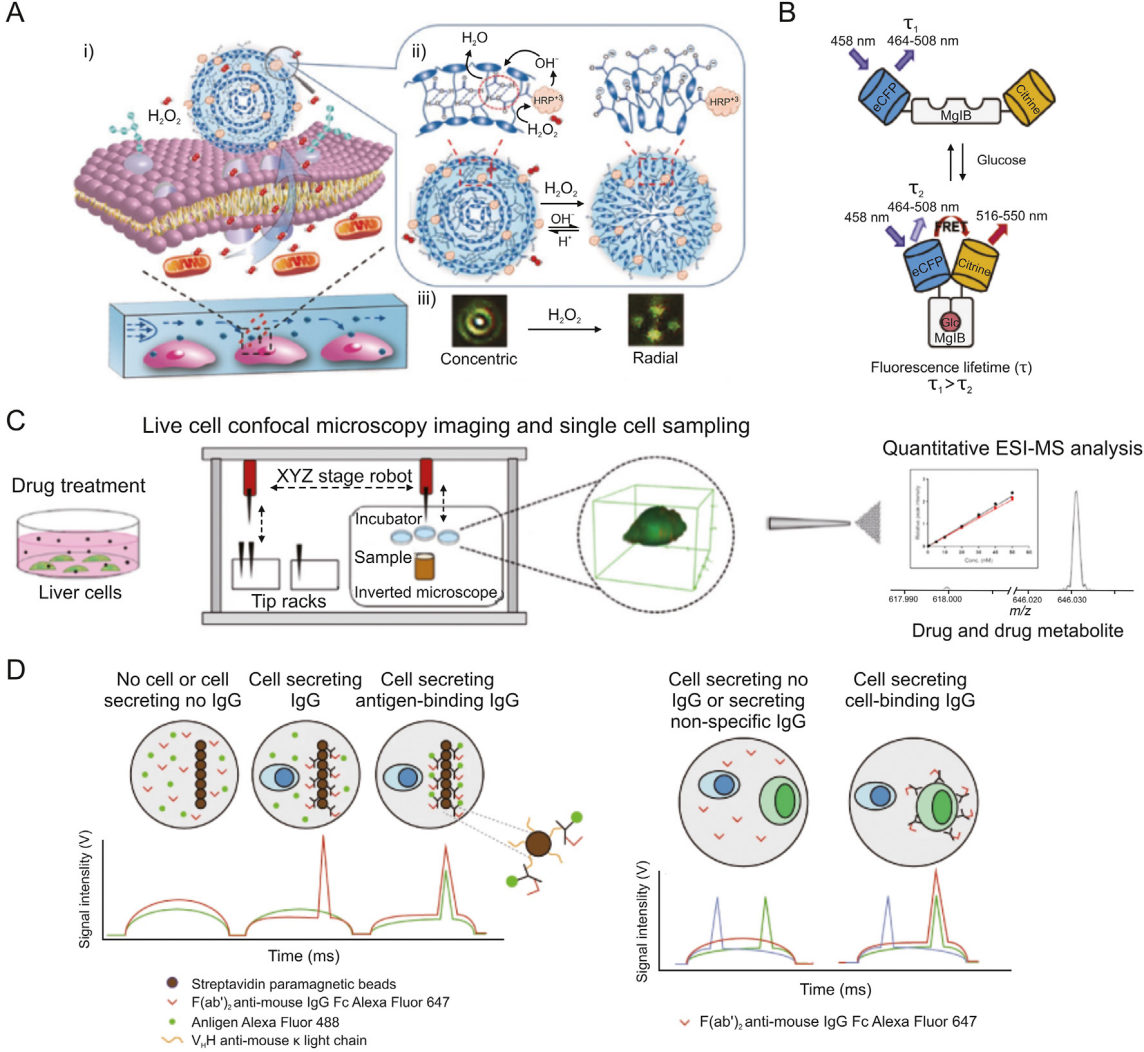
Optical imaging technology for single-cell metabolic detection. (A) Schematic diagram of the LCEM-HRP immobilized on the cell membranes and its transfiguration to detect released H_2_O_2_. Reprinted with permission from Ref. [47], copyright 2020 Wiley-VCH GmbH. (B) Schematic diagram of the conformational shift of the glucose fluorescence resonance energy transfer (FRET) biosensor when binding to intracellular glucose, which therefore reflected the intracellular glucose levels. Reprinted with permission from Ref. [50], copyright 2021 Cell Press. (C) Schematic diagram of drug response analysis of single cells by the combination of confocal microscopy and mass spectrometry. Reprinted with permission from Ref. [53], copyright 2020 American Chemical Society. (D) Schematic diagram of the fluorescent distribution in the droplets for screening active cell-secreting soluble antigens and the membrane antigens. Reprinted with permission from Ref. [55], copyright 2020 Springer Nature.

Single-cell molecular secretion is detected using optical imaging techniques. Li et al. [47] developed liquid crystal elastomer microspheres functionalized with horse-radish peroxidase (LCEM-HRP) in order to track hydrogen peroxide release on specific cell surfaces in real-time (Fig. 6A). These 2 μm LCEM-HRP microspheres could be directly fixed to the cell membrane without internalization. H_2_O_2_ produced by the cell was converted to OH^−^ by HRP, while the polarization morphology of LCEM-HRP switched from a Concentric to a Radial configuration. A linear relationship between the H_2_O_2_ concentration and the timing of the conformational shift was observed. This approach was high biocompatibility, sensitivity, cost-effectiveness, and rapid response. Wang et al. [48] developed a high-throughput, multiplexed single-cell protein secretion analysis system based on graphene oxide quantum dots (GOQDs). The technology included antibody barcode arrays to detect secreted biomarkers as well as microchambers for single-cell isolation and culture. Multiple secreted proteins were in situ detected quantitatively and sensitively by using fluorescence imaging. With the help of this platform, they were able to accurately measure 12 secreted biomarkers and identify five cell types with about 90% accuracy.

Additionally, optical imaging allows for continuously characterizing medication absorption and metabolism in single cells. Alshammari et al. [49] used spectral microscopy to characterize the dynamic process of doxorubicin (dox) distribution and its metabolism to doxorubicinol (dox'ol) within individual cells. Kondo et al. [50] utilized a glucose fluorescence resonance energy transfer (FRET) biosensor to dynamically track glucose levels in single cells. This allowed the identification of cellular metabolic states and revealed the regulation of the glycolytic pathway by phosphatidylinositol 3-kinase (PI3K) signaling (Fig. 6B). Additionally, optical imaging enables in vivo, three-dimensional, dynamic metabolic imaging of living tissues and organs [51,52], providing spatially resolved metabolic data.

Optical imaging is a non-destructive metabolic detection method that can be conjugated with other metabolic detection methods to gather multi-dimensional single-cell metabolic data. Pedro et al. [53] reported a combination of live-cell confocal microscopy imaging and high-resolution MS (Fig. 6C). They accurately quantified the drug amiodarone (AMIO) and its major metabolite, N-desethylamiodarone (NDEA), in individual cells. Through this method, they linked cellular drug concentrations to phospholipid levels and morphological characteristics. Additionally, to create multi-omics signaling networks at the single-cell level, optical imaging can be combined with DNA sequencing [54] and RNA sequencing. Gérard et al. [55] developed a high-throughput single-cell lgG-active secretory screening and sequencing system called CelliGO. Fluorescence-based intra-droplet antigen-antibody binding was used to separate primary plasma cells secreting active IgG, and single-cell barcode reverse transcription was used to sequence the V-genes of the paired antibodies (Fig. 6D). This method established the relationship between secretory IgG phenotype and antibody genotype and is applicable to the screening of both soluble antigens and membrane antigens.

#### 2.2.2 Raman spectroscopy

**Fig. 7:**
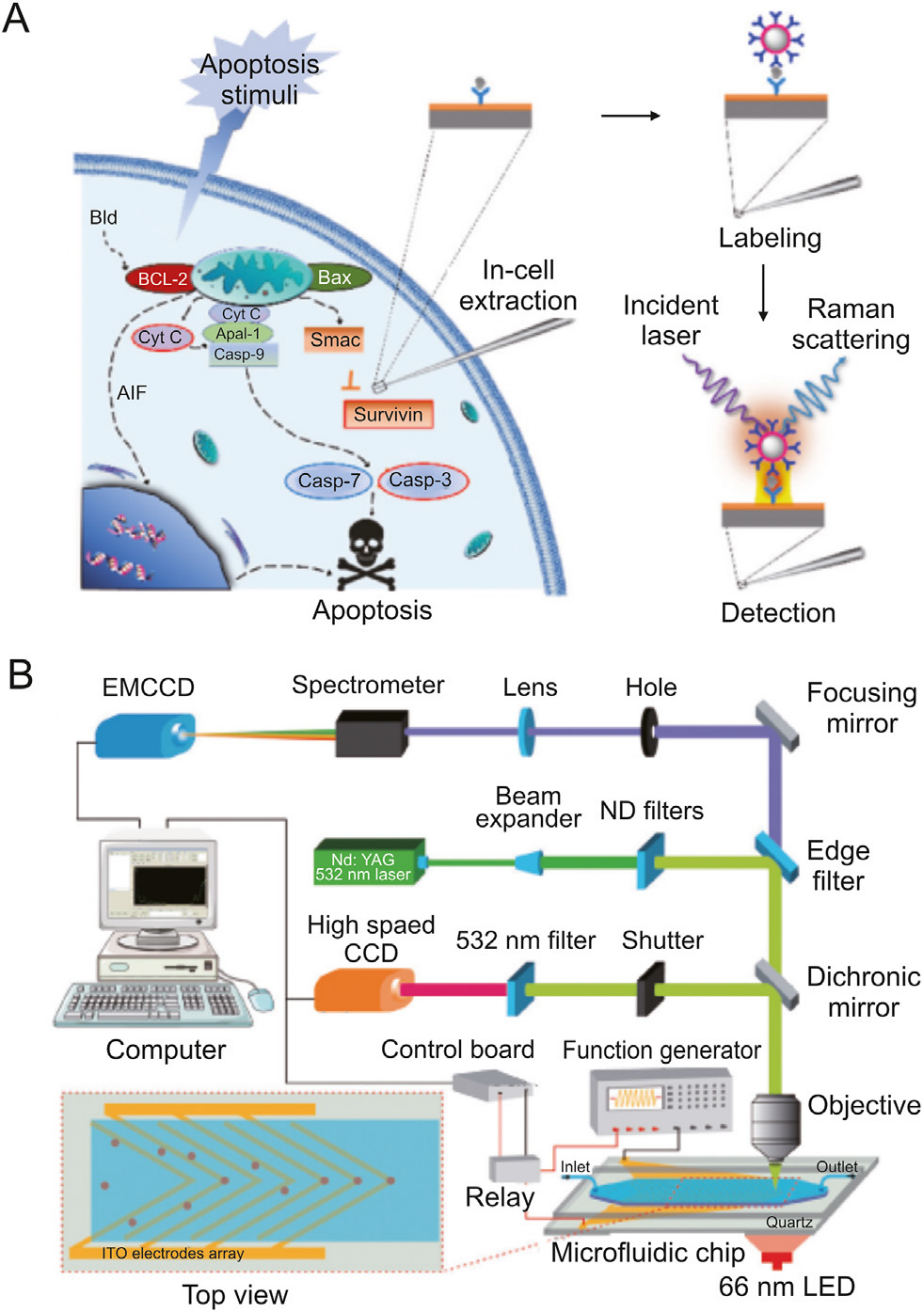
Raman spectroscopy for single-cell metabolic detection. (A) Schematic diagram of the scPISA method for signaling protein detection in living single cells. Reprinted with permission from Ref. [56], copyright 2020 American Chemical Society. (B) Schematic diagram of the Raman flow cytometry for high-throughput single-cell metabolic phenome profiling. Reprinted with permission from Ref. [63], copyright 2022 Wiley-VCH GmbH.

Surface-enhanced Raman scattering (SERS) has emerged as a promising detection technique for investigating single-cell metabolism. SERS provides highly sensitive detection of cellular metabolites in trace amounts by utilizing the plasmonic amplification of Raman signals on nanostructured metal surfaces. Wen et al. [56] introduced a single-cell Plasmonic ImmunoSandwich Assay (scPISA) that combined microinjection technology, plasmon-enhanced Raman scattering, and nanoparticle labeling (Fig. 7A). This method made it possible to analyze the dynamic interactions between caspase-3, cytochrome C, and survivin in tumor cells that had received anticancer medication [57].

Cellular metabolic activity has also been monitored using Raman spectroscopy. Wang et al. [58] used carbon-deuterium (C-D) bond characteristic Raman spectra to analyze metabolic changes caused by anticancer medication treatment in cells by culturing the cells with 30% heavy water. Zhao et al. [59] were able to quantify the activity of lipid and protein synthesis in single cells by using deuterium-labeled branched-chain amino acids (d-AA) and deuterium-labeled palmitic acid (d-PA). Chen et al. [60] created a collection of multiplex Raman probes that allowed for the assessment of cell surface proteins, endocytic activities, and metabolic dynamics under drug disruption.

SERS provides the potential for label-free multiplex detection based on spectral fingerprints, in contrast to fluorescence optical imaging. Li et al. [61] reported a Raman imaging technique for determining the activity of cytochrome P450 in living cells through redox-state and spin-state-sensitive measurements. Hsu et al. [62] established a label-free and noninvasive single-cell Raman microspectroscopy platform that coupled with machine learning data analysis. They were able to identify the biomarkers that set hiPSCs apart from other neuronal derivatives.

SERS also offers noninvasive and high-throughput detection approaches. In order to concentrate single cells and quickly capture their dynamic metabolic features, Wang et al. [63] developed a robust Raman flow cytometry method using periodic positive dielectrophoresis-induced deterministic lateral displacement (pDEP-DLD) force, achieving an analysis efficiency of 2,700 events/min (Fig. 7B).

#### 2.2.3 Electrochemical detection

**Fig. 8:**
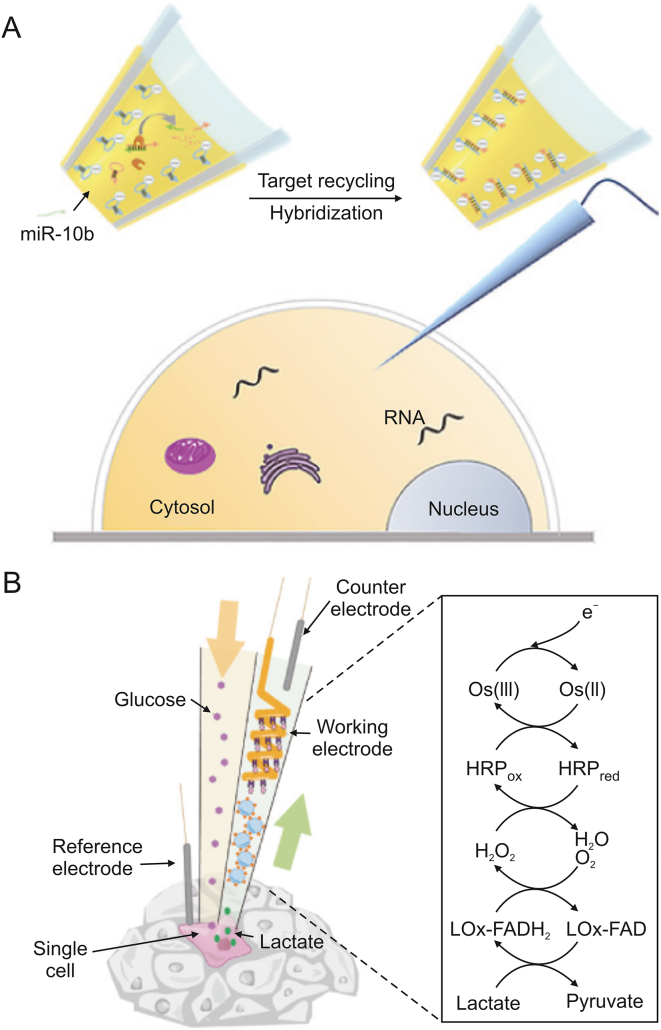
Electrochemical detection for single-cell metabolic detection. (A) Schematic diagram of the nanopipette for single-cell miRNA-10b detection. Reprinted with permission from Ref. [64], copyright 2021 Wiley-VCH GmbH. (B) Schematic diagram of the push-pull nozzle for in situ single-cell stimulation and lactate detection. Reprinted with permission from Ref. [67], copyright 2021 American Chemical Society.

Electrochemical detection is a less invasive and cell-preserving technique for studying single cells. It involves inserting a conductive nanopipette into the cell's interior. Target intracellular molecules can be examined by measuring the electro-physiological changes of the target molecules or their interaction with functionalized molecules in the probe. Wang et al. [64] introduced an electrochemical method for detecting the amounts of RNA expression in single cells. For the purpose of identifying and capturing the target RNA, hairpin DNA was previously formed on the inner wall of the nanopipette (Fig. 8A). RNA amplification and signal production were accomplished using catalytic hairpin assembly (CHA) and duplex-specific nuclease (DSN). This technique was used to categorize breast cancer cells according to the level of miR-10b expression and investigate the effects of drug therapy. Xu et al. [65] developed a method for detecting the intracellular amounts of the amino acid L-His. based on the L-His-dependent cleavage of DNAzyme. The probe also showed how L-His was distributed spatially within cells and how it responded to drugs in a time-dependent manner. Vaneev et al. [66] used an electrochemical probe to monitor the real-time generation of reactive oxygen species (ROS) induced by anticancer drug therapy in mice. They also evaluated the variation in ROS levels at various tumor depths.

Zhou et al. [67] suggested an in-situ push-pull electrochemical probe for single-cell stimulation and real-time detection that is distinct from extractive electrochemical probes (Fig. 8B). This device extracted fluid from one side of the cell while supplying it with a glucose solution. By employing horseradish peroxidase (HRP)-conjugated redox polymers, the system enabled the real-time quantification and analysis of lactate secretion.

#### 2.2.4 RNA sequencing

scRNA-seq is the most commonly used technology to reveal cellular transcriptome heterogeneity. In 2015, Macosko et al. [68] reported a highly parallel and high-throughput method for single-cell whole-genome expression analysis by co-encapsulating barcoded gel beads with single cells in droplets. There have been a number of scRNA-seq commercial instruments widely employed to investigate rare drug-resistant cell populations and optimize treatment strategies due to their universal application and adaptability. However, existing scRNA-seq techniques have several limitations: they only capture a portion of transcripts, lack temporal information due to their cell-destructive feature, and do not provide spatial information. As a result, recent developments have concentrated on developing scRNA-seq methodologies with temporal and spatial resolution, as well as optimizing full-length transcriptome profiling.

Tian et al. [69] created a computational framework that enables a single-cell full-length investigation of mutations and splicing by combining high-throughput short-read sequencing with 10%–20% subsample long-read sequencing. Comprehensive single-cell full-length sequencing in people and mice was investigated with this technique, which also made it easier to find isoforms, analyze splicing patterns, and detect mutations. Salmen et al. [70] employed RNA fragmentation, end repair, and poly(A) tailing to capture both non-polyadenylated and polyadenylated transcripts in single cells. This enabled high-throughput and cost-effective single-cell total RNA sequencing.

**Fig. 9:**
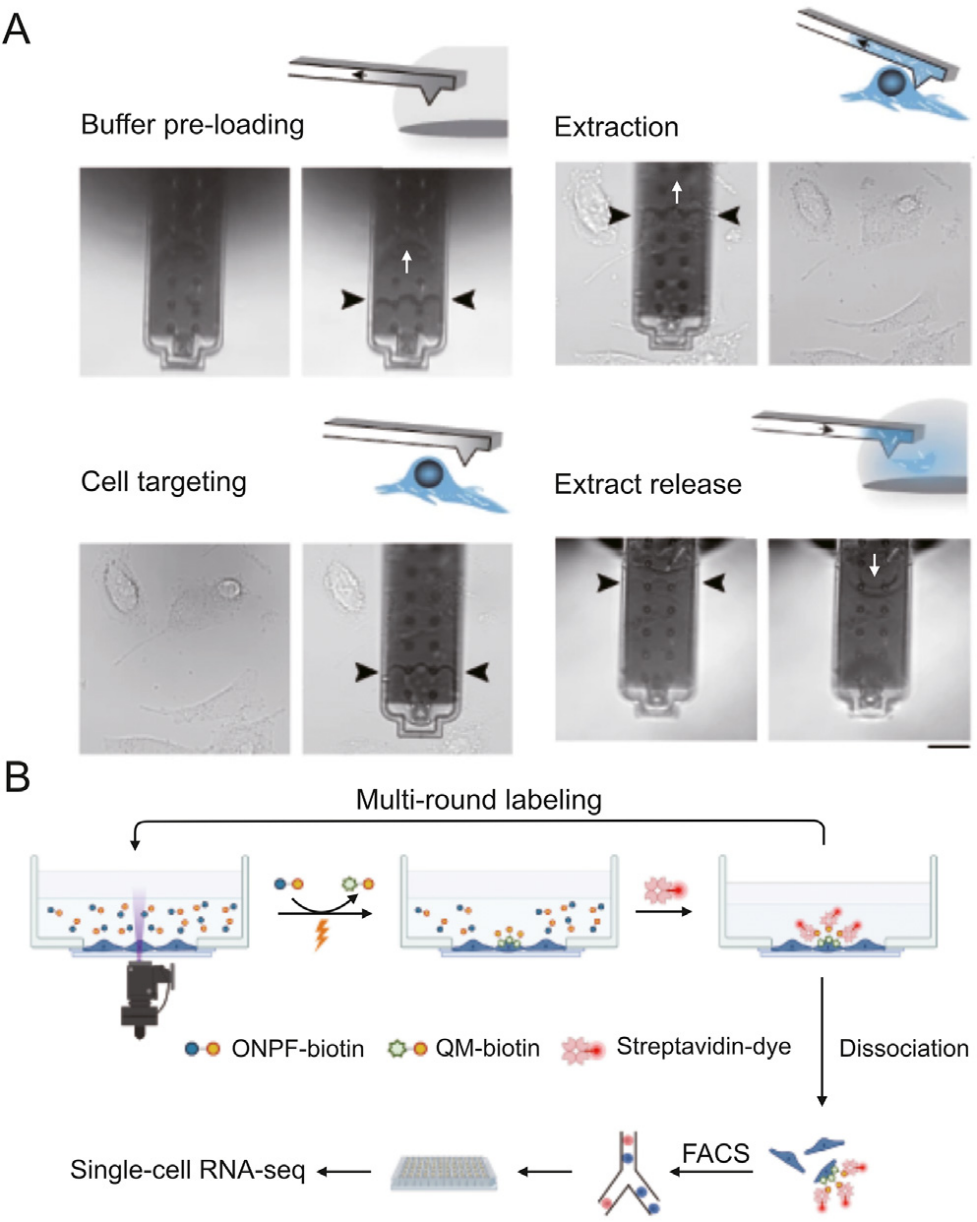
RNA sequencing for single-cell metabolic detection. (A) Schematic diagram of the Live-seq method for single-cell transcriptome profiling while preserving cell viability using fluidic force microscopy. Reprinted with permission from Ref. [72], copyright 2022 Springer Nature. (B) Schematic diagram of the optical cell tagging for spatially resolved scRNA-seq. Reprinted with permission from Ref. [74], copyright 2022 Wiley-VCH GmbH.

Qiu et al. [71] developed a method for the parallel analysis of newly generated and preexisting mRNAs in the same cell to study temporal RNA dynamics caused by pharmacological treatment. To identify newly transcribed RNA, they used exogenous nucleoside analogs called 4-thiouridine (4sU), which were then chemically converted into cytidine analogs for detection in reverse-transcribed RNA sequencing. A single-cell sequencing technique was created by Chen et al. [72] to constantly track the transcriptional changes occurring in the same cell while maintaining cell viability and functionality (Fig. 9A). By using fluidic force microscopy in combination with low-damage cytoplasmic biopsy, they were able to overcome the limitations of using scRNA sequencing as an end-point analysis method.

Meyer et al. [73] utilized single-cell sequencing, in situ single-cell separation, and live-cell imaging to demonstrate the association between spatial information and the transcriptome. Following drug administration, they used fluorescence imaging to monitor cell behavior during drug stimulation and make cell fate predictions. In situ cell extraction was then carried out using laser-capture microdissection, followed by single-cell RNA sequencing. This technique made it possible to record dynamic cellular reactions to medications before sequencing and to find non-heritable drug resistance mechanisms. Tang et al. [74] developed Optical Cell Tagging, a method for designating cell position information (Fig. 9B). UV irradiation converted ONPF-biotin into QM-biotin to achieve covalent fluorescent labeling of specific cells. Fluorescence-assisted cell sorting (FACS) and scRNA-seq were then employed for sequencing the selected cells. Vahid et al. [75] constructed tissue cartography at single-cell resolution by mapping single-cell RNA sequencing profiles to the spatial expression profiles. This combination addressed limitations in spatial transcriptomics methodologies, including limited gene recovery, low spatial resolution, and the absence of spatial information.

#### 2.2.5 MS

Due to its benefits of high sensitivity, label-free, and simultaneous detection of several metabolites, MS technology is regarded as a highly promising method for single-cell metabolite detection. MS-based single-cell metabolomics analysis methods have evolved significantly in recent years. Examples include nano-electrospray ionization mass spectrometry (nano-ESI-MS), mass cytometry, and MS imaging. These techniques provide high throughput, spatially resolved metabolite detection, and great coverage of metabolic molecules [76]. In the study of drug metabolism, MS detection allows for the monitoring of drug uptake, metabolism, and elimination within individual cells as well as the assessment of protein and metabolic changes in response to drug perturbation.

**Fig. 10:**
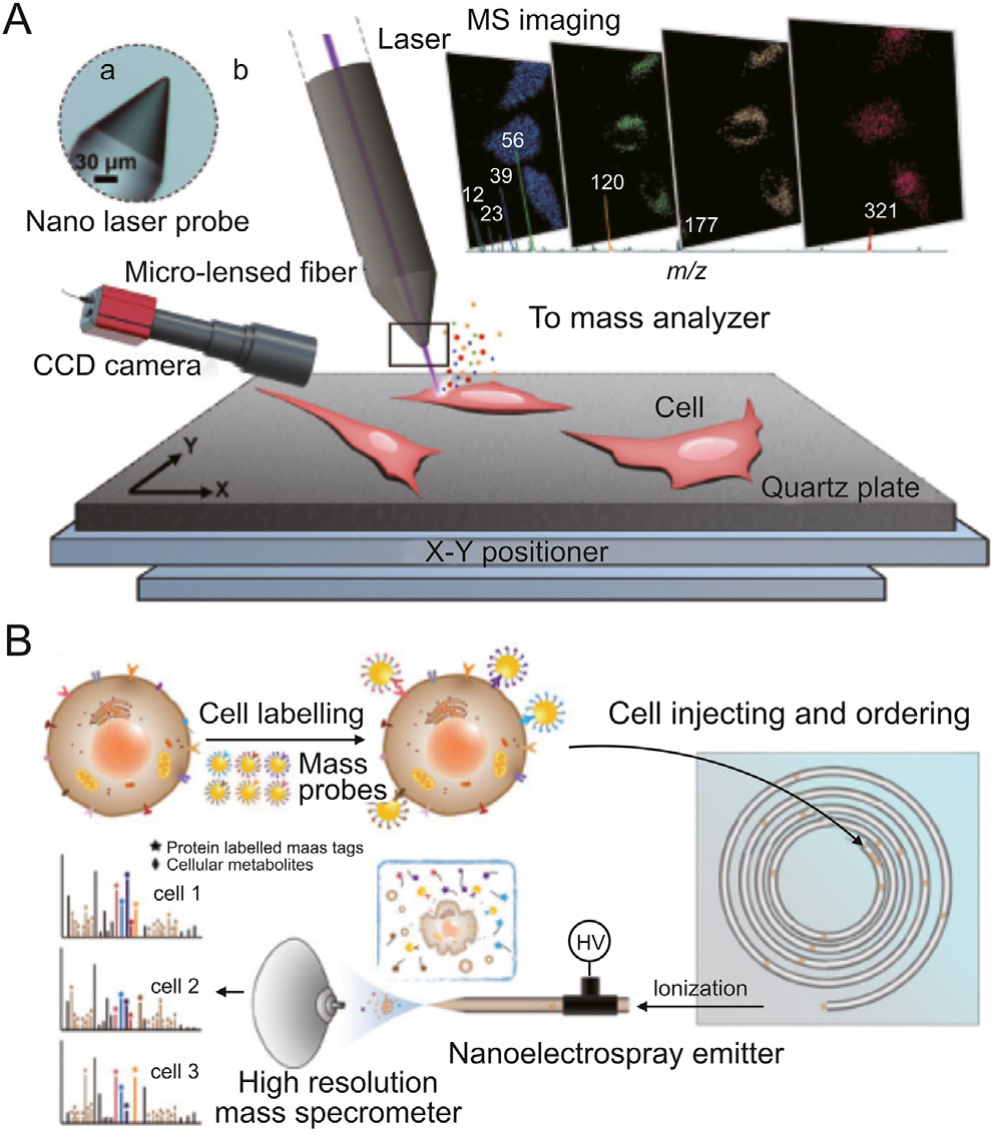
Mass spectrometry for single-cell metabolic detection. (A) Schematic diagram of the nano laser probe-based MS imaging for drug distribution profiling of single cells. Reprinted with permission from Ref. [79], copyright 2020 Wiley-VCH GmbH. (B) Schematic diagram of the multi-dimensional organic MS cytometry for multiplexing detection of single-cell proteins and metabolites. Reprinted with permission from Ref. [88], copyright 2021 Wiley-VCH GmbH.

Using MS, drug metabolism mechanisms within a single cell have been studied. To track the cellular transformation of As (III), Men et al. [77] reported a single-cell metabolomics measurement approach that includes a microfluidic device for single-cell focusing, capillary electrophoresis, and inductively coupled plasma mass spectrometry (ICP-MS). This method allowed the investigation of As uptake efficiency, elimination kinetics, and the main arsenic species generated during cellular transformation and elimination in HepG2 cells. Lim et al. [78] used ICP-MS to track the accumulation of Pt-based metallodrugs in ovarian cancer cells. It showed a correlation between decreased intracellular Pt accumulation and cellular drug resistance. Information on the spatial distribution of drug molecules within cells was provided by MS imaging. Meng et al. [79] developed a micro-lensed fiber lasers-based desorption MS imaging technique to visualize the subcellular distribution of small-molecule drugs (Fig. 10A). This label-free method, with a lateral resolution of 300 nm, was employed to monitor the dynamic process of anticancer drug released from nanocarriers within lysosomes and then entered into the cell nucleus and finally induced cell apoptosis. A near-field laser desorption/laser postionization mass spectrometry imaging approach was developed by Cheng et al. [80] and is capable of distinguishing the subcellular distribution of two structurally similar acridine drugs.

In order to study the intracellular metabolic changes under drug perturbation, Jia et al. [81] developed a protein imaging technique that combined time-of-flight secondary ion mass spectrometry (TOF-SIMS) with genetically encoded chemical markers. They studied the interactions of cisplatin, DNA binding proteins, and DNA damage by labeling target proteins with fluorinated non-natural amino acids. Végvári et al. [82] developed an integrated MS approach using fluorescence-activated cell sorting, tandem mass tag (TMT) isobaric labeling, and carrier proteome analysis to investigate protein dynamics in drug treatment response. Brunner et al. [83] significantly improved proteomics detection sensitivity by combining sub-μL sample preparation, very low flow chromatography, and trapped ion mobility mass spectrometry. They obtained a remarkable data fidelity of 92%.

MS has also been used to examine small metabolic chemical alterations that occur during drug therapy. Zhu et al. [84] employed intact living-cell electro-launching ionization MS for the label-free investigation of drug mechanisms at the single-cell level. They described how gefitinib affected a number of metabolic pathways, including those for glycerophospholipid, sphingolipid, and arginine and proline metabolism. Hiyama et al. [85] reported a live single floating cell metabolomics detection method based on mass spectrometry detection and nanospray tip sampling. From patient-derived CTC cells, the technique was able to extract crucial molecular data, including more than 300 lipids, amino acids, catecholamine metabolites, etc., as well as the medication streptomycin for which the patient was receiving therapy. This provided a method of evaluating drug absorption by liquid biopsy. Ali et al. [86] combined this technique with 3D holographic and tomographic laser microscopy for sampling and quantitative analysis of subcellular contents. Cuypers et al. [87] developed a matrix-assisted laser desorption/ionization-mass spectrometry imaging method capable of rapid cell classification for breast cancer tissue sections. It provided information on metabolites and lipid abundances in the *m*/*z* 200–1200 range. Xu et al. [88] introduced a multi-dimensional organic mass spectrometry technique based on nanoelectrospray ionization mass spectrometry (Fig. 10B). It enabled the simultaneous quantitative measurement of about 100 metabolites and six surface proteins in a single cell. For the conversion of protein signals, they used rhodamine-based mass tags and specific antibodies assembled on gold nanoparticles. The protein and small molecular metabolites were then analyzed simultaneously. This technique is used to assess metabolic variations in MCF-7 cells with various phenotypes after doxorubicin treatment.

## 3 Applications of single-cell metabolic analysis in pharmacological research

### 3.1 Drug response and resistance pathway

Cellular heterogeneity leads to distinct metabolic responses among different cell types within a lesion upon drug administration. When a medication stimulates a lesion, cellular heterogeneity causes various cell types to respond differently metabolically. Single-cell research techniques uncover complex medication effectiveness mechanisms, laying a solid theoretical foundation for therapeutic treatments. Additionally, they made it possible to identify cell subtypes that are extremely drug-sensitive or drug-resistant, providing novel approaches to therapy. Single-cell analysis has been useful in assessing the therapeutic effects of medications at many levels, including cell lines, animal models, and patients. This method offers insights into pharmacological mechanisms and aids in the creation of innovative therapeutics (Table 3) [[89], [90], [91], [92], [93], [94], [95], [96], [97]].

**Table 3:** Single cell metabolic analysis for drug response and resistance.

Sample	Drug	Method	Results	Refs.
HCT116	Decitabine	scRNA-seq	The mechanisms of the efficacy of low-dose DNA demethylating therapy: complete demethylation of specific cancer-related genes, such as cell cycle regulation, WNT pathway, p53 pathway, and TGF-β pathway.	[89]
RKO, HCT116, SW480	5-fluorouracil, camptothecin, etoposide	scRNA-seq	DNA-damage-induced gene expression and the corresponding cell-fate responses.	[90]
Rat kidney	Losartan	scRNA-seq, ST seq	Spatial heterogeneity of drug responses of different glomeruli in the kidney.	[91]
Primary striatal neurons from Rat pups	SKF 81297 and/or NMDA, forskolin	FRET biosensor imaging	PKA and ERK1/2 responses to pharmacological stimulation and novel interactions between PKA and ERK1/2 signaling.	[92]
Lymph nodes from experimental autoimmune uveitis mouse	Prednisone	Flow cytometric analysis, scRNA-seq	Prednisone induces anti-inflammatory effects through modulation of immune cell composition by affecting a variety of cell type-specific and non-specific genes, including *Hsp90aa1, Id3*, *Cxcl2*, etc.	[93]
Neonatal mouse ovarie	Müllerian inhibiting substance	scRNA-seq	MIS may be a potent ovarian inhibitor that inhibits follicle development by suppressing cell proliferation, triggering granulosa cell quiescence, and preventing granulosa cell differentiation.	[94]
Tumor sample from ovarian cancer patients	Niraparib and pembrolizumab	Immunogenomic profiling and highly multiplexed single-cell imaging	Two new candidate predictive mutation biomarkers, signature 3 and positive immune score, were identified as determinants of treatment response.	[95]
Peripheral blood mononuclear cells form PD-L1-negative patients	Pembrolizumab	scRNA-seq	The heterogeneity of immune cells before and after treatment was revealed. And identified predictive markers to Pembrolizumab treatment, such as ID2, PIK3CD, UQCR10, etc.	[96]
OA chondrocytes from knee arthroplasty patients	BMS-345541 and kartogenin	Time-of-flight mass cytometry	The two drugs screened in the animal model showed a differential response in human treatment, with BMS-345541 eliciting a uniform drug response in all patients and only a minority responding to kartogenin.	[97]

scRNA-seq: single-cell sequencing; ST seq: spatial transcriptome sequencing; NMDA: *N*-methyl-D-aspartic acid; FRET: forster resonance energy transfer; PKA: protein kinase A; ERK1/2: extracellular signal-regulated kinase 1/2; MIS: Müllerian inhibiting substance; PD-L1: programmed cell death 1 ligand 1; ID2: inhibitor of DNA binding 2; OA: osteoarthritis.

Takeshima et al. [89] used scRNA-seq technology to investigate the effects of low-dose decitabine-based DNA demethylation treatment on HCT116 cells. They discovered that some cancer-related pathways, such as cell cycle regulation, the WNT system, the p53 pathway, and the TGF pathway, displayed complete demethylation of the associated genes, despite only finding a minor reduction of about 7.8% in DNA methylation levels among the cell population. As a result, the cell cycle returned to normal and cellular senescence was induced. Onodo et al. [91] employed spatial transcriptome sequencing (ST-seq) and scRNA-seq to perform spatial transcriptome analysis on rat kidneys. The diverse response of various cell types to kidney injury brought on by losartan was investigated. They discovered *Ren* expression to be a marker of the chlorothiazide response and noted regional variability in the response across renal glomeruli.

Besides, single-cell analysis has been useful in the development of potential new therapeutic medicines. Meinsohn et al. [94] investigated the role of Müllerian inhibiting substance (MIS) in antagonizing primordial follicle activation. They demonstrated through scRNA-seq testing that MIS was a promising ovarian suppressor because it inhibited cell proliferation, caused granulosa cell quiescence, and blocked granulosa cell differentiation.

Patient samples have been analyzed using single-cell analysis in order to better understand treatment efficacy. Färkkilä et al. [95] studied tumor samples from ovarian cancer patients receiving niraparib and pembrolizumab therapy using immunogenomic profiling and highly multiplexed single-cell imaging. They discovered Signature 3 and positive immune score as two new candidate predictive mutational biomarkers that are essential for therapeutic response.

A significant factor in disease recurrence and treatment resistance is tumor heterogeneity. The discovery of specific resistance mechanisms and the identification of resistant cell subtypes are made possible by single-cell analysis technology. Kashima et al. [98] used scRNA-seq and single-cell transposase-accessible chromatin sequencing (scATAC-seq) to examine the resistance of tyrosine kinase inhibitors in lung cancer cell models and clinical samples with epidermal growth factor receptor (EGFR) mutations. They discovered the brand-new, essential resistance gene *CD74* and showed that its overexpression drives osimertinib resistance by preventing apoptosis and promoting tumor regeneration. At the single-cell level, Taavitsainen et al. [99] discovered pre-existing and treatment-enduring cell subtypes linked to enzalutamide resistance in prostate cancer, which corresponded with cancer recurrence. Cohen et al. [100] compared individuals with primary refractory multiple myeloma with those who had already experienced an early relapse, discovering various resistance pathways such as hypoxia tolerance, protein folding, and mitochondrial respiration. They also identified peptidylprolyl isomerase A as a new target for the treatment of multiple myeloma that is resistant to current therapies.

### 3.2 Therapeutic target discovery

Research on single-cell metabolism also identifies and verifies potential therapeutic targets. New potential gene or protein targets can be found in the unknown cell phenotypes and biomarkers. Additionally, it is demonstrated how signaling pathways are activated and compensated both inside and between cells, laying the theoretical groundwork for successful combination therapy. Overall, single-cell analysis is a useful method in drug development because it provides unique therapeutic approaches and prospective pharmacological targets for diverse malignancies (Table 4) [[101], [102], [103], [104], [105], [106]].

**Table 4:** Single cell metabolic analysis for therapeutic target discovery.

Sample/disease	Method	Treatment pathways	Candidate drug delivery targets	Refs.
Lung tumor endothelial cell from human and murine	scRNA-seq, Time-of-flight mass cytometry	Blocking tumor angiogenesis by targeting endothelial cells	Targeting collagen-modifying enzymes such as PLOD and LOX to inhibit vascular sprouting.	[101]
Bone marrow mononuclear cells of acute myeloid leukemia	scRNA-seq (Microwell-seq)	Targeting AML progenitor cells that are closely related to bone marrow cells in the genetic network	CCNA1 and RAB37 were only highly expressed in AML progenitor cell clusters, therefore, could be used as new drug targets.	[102]
Ovarian cancer	scRNA-seq	Targeting key prognostic phenotypes or markers that regulate OC progression	Paternally expressed gene 10 (PEG10) affects cancer stem cell self-renewal and promotes drug resistance through the NOTCH pathway. Therefore could be used as a new drug target.	[103]
Metastatic lung cancer cell	Time-of-flight mass cytometry	AXL-associated signaling pathways	The redundant signaling networks of AXL, TGFβ, and JAK1 together promote tumor growth and metastatic spread. Therefore combined inhibition is a new therapeutic strategy.	[104]
Renal cell carcinoma	Multiparameter flow cytometry, scRNA-seq	Reducing infiltration of polymorphonuclear MDSCs and TAMs to inhibit immune checkpoint inhibitor resistance in solid tumors	Anti-interleukin-1 beta (IL1β) was able to act complementarily with anti-PD-1 and was a candidate to overcome adaptive immune resistance.	[105]
Gastric cancer and paired normal tissue	- (single-cell data from the website)	Blocking metabolic reprogramming due to cancer cell-macrophage interactions in high stemness clusters	Cells in high stemness clusters exhibit high GAG metabolic signatures. High expression of SERPINE1 in cancer cells and HS6ST2 in macrophages are potential novel drug targets.	[106]

-: no data. scRNA-seq: single-cell sequencing; PLOD: procollagen-lysine, 2-oxoglutarate 5-dioxygenase; LOX: lysyl oxidase; AML: acute myeloid leukemia; OC: ovarian cancer; TGFβ: transforming growth factor β; MDSCs: myeloid-derived suppressor cells; TAMs: tumor-associated macrophages; GAG: glycosaminoglycan.

Goveia et al. [101] classified the endothelial cells from human, mouse, and lung tumor sources with scRNA-SEQ, discovering a distinct tip endothelial cell phenotype associated with migration and basement membrane remodeling, along with the specific marker genes. They noticed the collagen prolyl hydroxylase (PLOD) was upregulated, suggesting it as a possible therapeutic target for cancer treatment with an anti-angiogenesis strategy. Taverna et al. [104] used single-cell proteomic profiling to undertake a thorough analysis of the AXL kinase inhibitor TP-0903. They discovered that AXL inhibition caused the SMAD4/TGF- signaling to be suppressed and the JAK1-STAT3 signaling to be activated in response, offering new information about the complex oncogenic signaling network and pointing to a combination AXL-JAK1 inhibition as a viable treatment strategy. Sung et al. [106] investigated the effects on metabolic pathways of the interaction between tumor cells and macrophages. They discovered a metabolic reprogramming characterized by increased expression of *HS6ST2* in adenocarcinoma cells and *SERPINE1* in macrophages, with the help of the common ligand RPS2. This characteristic was linked to a poor prognosis in patients with gastric cancer, emphasizing the possibility of *HS6ST2* and *SERPINE1* as therapeutic targets.

### *3.3* *In vitro* disease model evaluation

In disease research and drug screening, two-dimensional cell culture models and animal models have limited physiological relevance, which hinders the understanding of pathogenic pathways and the efficiency of therapeutic development. Various in vitro three-dimensional models, including co-culture models, multicellular spheroid models, and organoids, have been created to solve this problem. However, evaluating the effectiveness of these models is challenging. A novel method of characterizing cellular heterogeneity, intricate interactions, and pharmacological responses is provided by single-cell analysis, which provides insight into how closely these models resemble actual diseases. Recently, scRNA-seq is a useful method for developing novel in vitro models and assessing their efficacy (Table 5) [[107], [108], [109], [110], [111], [112], [113]].

**Table 5:** Single cell metabolic analysis for drug delivery model evaluation.

Organ/Disease	Type of the model	Source	Method	Evaluation results	Refs.
Colorectal cancer	Co-culture model	HCT116, PBMCs	scRNA-seq	The model reconstructed the concurrent T cell-mediated antitumor response under nucleoside analogue trifuridine (FTD) treatment, as well as the immunosuppressive effects.	[107]
Lung cancer	Multicellular spheroid	Patient-derived lung cancer cells, CLS1, CAFs	scRNA-seq	In vitro reconstruction of the stemness phenotype of replicating CSCs and the stemness niche supported by CAFs and used for high-throughput drug screening.	[108]
Liver fibrosis	Multicellular spheroid	HepaRG, hTERT-HSC, THP-1	scRNA-seq	TGF-β1 induced a fibrotic phenotype in spheroids, as evidenced by a decrease in albumin expression and an increase in αSMA expression.	[109]
Midbrain	Organoid	iPSCs	scRNA-seq	The model had multiple subtypes of neurons, including dopaminergic, GABAergic, glutamatergic, serotonergic neurons, and exhibited spontaneous electrophysiological functions.	[110]
Hepatobiliary tumor	Organoid	Patient-derived tumor cells	scRNA-seq	The organoid contained inherent differences with cell cycle and epithelial expression, as well as CSC heterogeneity associated with chemoresistance.	[111]
Leigh syndrome	Organoid	Patient-derived iPSCs	scRNA-seq	Neural progenitor cells in the organoid are unable to control neuronal morphogenesis because of metabolic defects brought on by *SURF1* mutations.	[112]
Cancer	Xenograft	Patient-derived tumor cells	scRNA-seq	Murine viral genomic contamination was widespread in patient-derived xenografts and resulted in significant changes in cellular expression levels in xenografts.	[113]

scRNA-seq: single-cell sequencing.

Messner et al. [109] developed a multicellular spheroid model by co-culturing patient-derived lung cancer cells (CLS1) with cancer-associated fibroblasts (CAFs) to mimic liver microtissues in vitro. In the liver microtissues, stimulation with TGF-1 caused cell type-specific fibrotic features. Smits et al. [110] differentiated human induced pluripotent stem cells (iPSCs) to construct human midbrain organoids. They used scRNA-seq to identify various neuronal subtypes within the organoids and then used imaging and electrophysiology techniques to investigate interactions and spontaneous electrical activity among the neurons.

Effective in vitro models let us better understand the disease's causes and offer treatment strategies. Inak et al. [112] generated organoid models of Leigh syndrome, a rare mitochondrial disease, using patient-specific iPSCs and CRISPR/Cas9 technology. *SURF1* mutations resulted in neural progenitor cells sustaining a proliferative glycolytic state that hinders neuronal morphogenesis, according to scRNA-seq and multi-omics analyses. Based on this finding, *SURF1* gene enhancement and mitochondrial biogenesis were identified as potential intervention strategies promoting early neuronal morphogenesis. Yuan et al. [113] brought attention to the problem of mouse viral interference in patient-derived xenografts using scRNA-seq. They showed that mouse virus sequences were widely present in xenografts, generating substantial alterations in gene expression levels and adversely influencing the results of drug research when employing xenografts as models.

## 4 Current challenges and future perspectives

The use of single-cell analysis technologies has substantially increased our understanding of diseases and aided in the development of new medications and therapy strategies. Single-cell analysis technology has been used clinically to diagnose and treat various diseases, including tumors, immune system disorders, and neurological disorders. Their applications have demonstrated particular benefits in guiding clinical diagnosis of pathologies, selecting and evaluating therapeutic regimens, and monitoring tumor progression. For the detection of measurable residual disease (MRD) in post-treatment acute myeloid leukemia (AML) patients, targeted single-cell sequencing identified patients with a high risk of recurrence more precisely [114]. Single-cell lineage monitoring of cancer persister cells showed non-genetic resistance mechanisms under treatment stress and provided new ideas for preventing cancer recurrence [3]. A number of single-cell databases were established as well, containing cellular profiles for a variety of illnesses and treatments. They helped researchers swiftly integrate and analyze a large number of findings from earlier studies to generate important medical insights [115,116]. Additionally, there has been some initial success with the use of single-cell analysis technology in the pharmaceutical sector. Important applications included the selection of preclinical illness models, pharmacodynamic and pharmacokinetic evaluation, and cancer drug target screening [117]. During the COVID-19 outbreak, Xie and co-workers used a high-throughput single-cell sequencing approach to screen and identify extremely potent neutralizing immunoglobulins from recovering convalescent patients. And the outcomes served as the basis for developing a potent drug [118].

In the fields of biomedical and pharmaceutical research, single-cell analysis technology is expected to be used extensively. But there are still a number of challenges that need to be addressed. First of all, since the metabolites are trace but diverse in single cells, comprehensive examination techniques for the wide variety of metabolites in single cells have not yet been created. Existing optical analyses have restrictions on their necessity to increase sensitivity by precise labeling. And mass spectrometry can only detect abundant and ionization-efficient molecules. Secondly, the response of single cells to the external environment is highly dynamic. Thus, the timeliness of the single-cell separation and detection process is required. Data biases caused by effects on cell metabolism and activity in single-cell isolation and sample preparation are not negligible. Furthermore, cellular metabolism has distinct temporal and spatial properties that are lost in most detection methods. Mass spectrometry and RNA sequencing are two examples of current techniques that can only measure at a single time point since they damage cells. Spatial transcriptome analysis and mass spectrometry imaging techniques are able to visualize single-cell location information, but their spatial resolution and coverage need to be improved. To understand the connections between various parameters, such as metabolomics, proteomics, and transcriptomics, a signaling network for single-cell multi-omics is also desired. Although the coupling of some detection techniques can partially solve this problem, methods for simultaneous detection of multi-omics remain to be developed. Overall, single-cell analysis techniques are evolving toward high-throughput, high temporal-spatial resolution, and multi-omics integration.

In fact, just a few single-cell analysis techniques, mainly mass cytometry and scRNA-seq, have been used by researchers in pharmacological and therapy research. The common advantages of these techniques are high-throughput and user-friendly commercially available instruments. Therefore, in addition to technical optimization, automation of the experimental procedure to eliminate human error and operational thresholds is vital to advance the practical application of single-cell analysis. Furthermore, as compared to standard cellular analysis, single-cell metabolic analysis generates a large and complex amount of data. A vast amount of single-cell multi-omics data has been collected, but determining how to extract useful biological information from it remains a challenge. It will rely on machine learning and deep learning [119].

Overall, single-cell metabolic analysis techniques have progressed quickly in recent years, but their pharmacological applications are still in the early stages. Single-cell analytical techniques are proven to be essential for elucidating intricate pathogenic pathways and treating complicated diseases with multicellular etiologies, such as cancer. The automation of experimental procedures, the ease of data processing, and cost savings will boost the adoption of single-cell technologies. Clinicians will eventually be able to categorize patients using single-cell technologies and combine them with extensive data analytics to predict drug reactions, enabling effective precision medicine. This depends on the coordinated development of analytical technology, clinical medicine, computer science, and other fields.

## 5 Conclusion

Single-cell analysis provides an unprecedented resolution for characterizing tissue heterogeneity, intercellular interactions, and cellular metabolic changes under drug perturbation. It has the potential to replace conventional analytical techniques as a key tool in disease research and drug development. In this review, we give a summary of the most recent developments in single-cell analysis technologies and how they apply to drug metabolism and drug response. Microfluidic techniques, including droplet microfluidics, microarrays, open microfluidic probes, and digital microfluidics, serve as the foundation for precise manipulation and control of single cells and culture conditions. The commonly used approaches for single-cell metabolic detection include optical imaging, Raman spectroscopy, electrochemical techniques, scRNA-seq, and mass spectrometry. Each has their advantages and limitations. Single-cell metabolic analysis is currently proceeding toward high-throughput, little cell damage, and simultaneous detection of numerous components. Furthermore, the application of single-cell analysis technology in studying drug response, drug resistance, disease research, new target discovery, and drug model evaluation is gaining considerable attention.

In conclusion, despite the remaining obstacles, single-cell analysis technology has great potential for widespread use in pharmaceutical research, given its advantages and further technological developments.

## CRediT author statement

**Ying Hou**: Investigation, Visualization, Writing - Original draft preparation, Reviewing and Editing; **Hongren Yao**: Visualization, Writing - Original draft preparation, Reviewing and Editing; **Jin-Ming Lin**: Funding acquisition, Conceptualization, Supervision, Project administration, Writing - Reviewing and Editing.

## Declaration of competing interest

The authors declare that there are no conflicts of interest.

## References

[1] Zenobi R. (2013). Single-cell metabolomics: Analytical and biological perspectives. Science.

[2] Altschuler S.J., Wu L.F. (2010). Cellular heterogeneity: Do differences make a difference?. Cell.

[3] Oren Y., Tsabar M., Cuoco M.S. (2021). Cycling cancer persister cells arise from lineages with distinct programs. Nature.

[4] Wheeler A.M., Eberhard C.D., Mosher E.P. (2023). Achieving a deeper understanding of drug metabolism and responses using single-cell technologies. Drug Metab. Dispos..

[5] Zhang Y., Chen S., Fan F. (2023). Neurotoxicity mechanism of aconitine in HT22 cells studied by microfluidic chip-mass spectrometry. J. Pharm. Anal..

[6] Ma S., Wu J., Liu Z. (2023). Quantitative characterization of cell physiological state based on dynamical cell mechanics for drug efficacy indication. J. Pharm. Anal..

[7] Chen T., Huang C., Wang Y. (2022). Microfluidic methods for cell separation and subsequent analysis. Chin. Chem. Lett..

[8] Lin X., Su J., Zhou S. (2022). Microfluidic chip of concentration gradient and fluid shear stress on a single cell level. Chin. Chem. Lett..

[9] Jiao Y., Gao L., Ji Y. (2022). Recent advances in microfluidic single-cell analysis and its applications in drug development. Trac Trends Anal. Chem..

[10] Ai Y., Zhang F., Wang C. (2019). Recent progress in lab-on-a-chip for pharmaceutical analysis and pharmacological/toxicological test. Trac Trends Anal. Chem..

[11] Zhong Q., Huang X., Zhang R. (2022). Optical sensing strategies for probing single-cell secretion. ACS Sens..

[12] Wallace G.Q., Masson J.F. (2020). From single cells to complex tissues in applications of surface-enhanced Raman scattering. Analyst.

[13] Yang Q., Huang X., Gao B. (2023). Advances in electrochemiluminescence for single-cell analysis. Analyst.

[14] Lei Y., Tang R., Xu J. (2021). Applications of single-cell sequencing in cancer research: Progress and perspectives. J. Hematol. Oncol..

[15] Tajik M., Baharfar M., Donald W.A. (2022). Single-cell mass spectrometry. Trends Biotechnol..

[16] Wu Z., Lawrence P.J., Ma A. (2020). Single-cell techniques and deep learning in predicting drug response. Trends Pharmacol. Sci..

[17] Shembekar N., Chaipan C., Utharala R. (2016). Droplet-based microfluidics in drug discovery, transcriptomics and high-throughput molecular genetics. Lab Chip.

[18] Hou Y., Chen S., Zheng Y. (2023). Droplet-based digital PCR (ddPCR) and its applications. Trac Trends Anal. Chem..

[19] Xu D., Zhang W., Li H. (2023). Advances in droplet digital polymerase chain reaction on microfluidic chips. Lab Chip.

[20] Yue X., Fang X., Sun T. (2022). Breaking through the Poisson Distribution: A compact high-efficiency droplet microfluidic system for single-bead encapsulation and digital immunoassay detection. Biosens. Bioelectron..

[21] Zhong J., Liang M., Tang Q. (2023). Selectable encapsulated cell quantity in droplets via label-free electrical screening and impedance-activated sorting. Mater. Today Bio..

[22] Zhang W., Li N., Lin L. (2020). Concentrating single cells in picoliter droplets for phospholipid profiling on a microfluidic system. Small.

[23] Zhang W., Li N., Lin L. (2021). Metabolism-based capture and analysis of circulating tumor cells in an open space. Anal. Chem..

[24] Wu W., Zhang S., Zhang T. (2021). Immobilized droplet arrays in thermosetting oil for dynamic proteolytic assays of single cells. ACS Appl. Mater. Interfaces.

[25] Xie T., Zhang Q., Zhang W. (2022). Inkjet-patterned microdroplets as individual microenvironments for adherent single cell culture. Small.

[26] Wong S.W., Lenzini S., Bargi R. (2020). Controlled deposition of 3D matrices to direct single cell functions. Adv. Sci..

[27] Ha B., Kim T.J., Moon E. (2021). Flow radiocytometry using droplet optofluidics. Biosens. Bioelectron..

[28] Zhou Y., Yu Z., Wu M. (2023). Single-cell sorting using integrated pneumatic valve droplet microfluidic chip. Talanta.

[29] Agnihotri S.N., Ugolini G.S., Sullivan M.R. (2022). Droplet microfluidics for functional temporal analysis and cell recovery on demand using microvalves: Application in immunotherapies for cancer. Lab Chip.

[30] Khajvand T., Huang P., Li L. (2021). Interfacing droplet microfluidics with antibody barcodes for multiplexed single-cell protein secretion profiling. Lab Chip.

[31] Radfar P., Ding L., de la Fuente L.R. (2023). Rapid metabolomic screening of cancer cells via high-throughput static droplet microfluidics. Biosens. Bioelectron..

[32] Xie R., Liu Y., Wang S. (2023). Combinatorial perturbation sequencing on single cells using microwell-based droplet random pairing. Biosens. Bioelectron..

[33] Zhang M., Zou Y., Xu X. (2020). Highly parallel and efficient single cell mRNA sequencing with paired picoliter chambers. Nat. Commun..

[34] Lin S., Yin K., Zhang Y. (2023). Well-TEMP-seq as a microwell-based strategy for massively parallel profiling of single-cell temporal RNA dynamics. Nat. Commun..

[35] Zhang Q., Feng S., Lin L. (2021). Emerging open microfluidics for cell manipulation. Chem. Soc. Rev..

[36] Mao S., Zhang W., Huang Q. (2018). *In situ* scatheless cell detachment reveals correlation between adhesion strength and viability at single-cell resolution. Angew. Chem. Int. Ed..

[37] Mao S., Zhang Q., Li H. (2018). Measurement of cell–matrix adhesion at single-cell resolution for revealing the functions of biomaterials for adherent cell culture. Anal. Chem..

[38] Mao S., Zhang Q., Li H. (2018). Adhesion analysis of single circulating tumor cells on a base layer of endothelial cells using open microfluidics. Chem. Sci..

[39] Zhang Q., Mao S., Li W. (2020). Microfluidic adhesion analysis of single glioma cells for evaluating the effect of drugs. Sci. China Chem..

[40] Zhang Q., Mao S., Khan M. (2019). *In situ* partial treatment of single cells by laminar flow in the “open space”. Anal. Chem..

[41] Zhang Q., Feng S., Li W. (2021). *In situ* stable generation of reactive intermediates by open microfluidic probe for subcellular free radical attack and membrane labeling. Angew. Chem. Int. Ed..

[42] Huang Q., Mao S., Khan M. (2020). Single-cell identification by microfluidic-based *in situ* extracting and online mass spectrometric analysis of phospholipids expression. Chem. Sci..

[43] Yi X., Zhang Q., Xie T. (2023). Microfluidic mixer for *in situ* ammonia analysis of single cells in mass spectrometry. Anal. Chem..

[44] Lamanna J., Scott E.Y., Edwards H.S. (2020). Digital microfluidic isolation of single cells for-Omics. Nat. Commun..

[45] Zhang Q., Xu X., Lin L. (2022). Cilo-seq: Highly sensitive cell-in-library-out single-cell transcriptome sequencing with digital microfluidics. Lab Chip.

[46] Xu X., Lin L., Yang J. (2022). Simultaneous single-cell genome and transcriptome sequencing in nanoliter droplet with digital microfluidics identifying essential driving genes. Nano Today.

[47] Li W., Khan M., Lin L. (2020). Monitoring H_2_O_2_ on the surface of single cells with liquid crystal elastomer microspheres. Angew. Chem. Int. Ed..

[48] Wang C., Wang C., Wu Y. (2022). High-throughput, living single-cell, multiple secreted biomarker profiling using microfluidic chip and machine learning for tumor cell classification. Adv. Healthcare Mater..

[49] Alshammari Q.A., Pala R., Katzir N. (2021). Label-free spectral imaging to study drug distribution and metabolism in single living cells. Sci. Rep..

[50] Kondo H., Ratcliffe C.D.H., Hooper S. (2021). Single-cell resolved imaging reveals intra-tumor heterogeneity in glycolysis, transitions between metabolic states, and their regulatory mechanisms. Cell Rep..

[51] Dawson C.A., Mueller S.N., Lindeman G.J. (2021). Intravital microscopy of dynamic single-cell behavior in mouse mammary tissue. Nat. Protoc..

[52] Heaton A.R., Rehani P.R., Hoefges A. (2023). Single cell metabolic imaging of tumor and immune cells in vivo in melanoma bearing mice. Front. Oncol..

[53] Pedro L., Rudewicz P.J. (2020). Analysis of live single cells by confocal microscopy and high-resolution mass spectrometry to study drug uptake, metabolism, and drug-induced phospholipidosis. Anal. Chem..

[54] Altemose N., Maslan A., Rios-Martinez C. (2020). μDamID: A microfluidic approach for joint imaging and sequencing of protein-DNA interactions in single cells. Cell Syst..

[55] Gérard A., Woolfe A., Mottet G. (2020). High-throughput single-cell activity-based screening and sequencing of antibodies using droplet microfluidics. Nat. Biotechnol..

[56] Wen Y., Liu J., He H. (2020). Single-cell analysis of signaling proteins provides insights into proapoptotic properties of anticancer drugs. Anal. Chem..

[57] Liu J., He H., Xie D. (2021). Probing low-copy-number proteins in single living cells using single-cell plasmonic immunosandwich assays. Nat. Protoc..

[58] Wang J., Lin K., Hu H. (2021). *In vitro* anticancer drug sensitivity sensing through single-cell Raman spectroscopy. Biosensors.

[59] Zhao Z., Chen C., Xiong H. (2020). Metabolic activity phenotyping of single cells with multiplexed vibrational probes. Anal. Chem..

[60] Chen C., Zhao Z., Qian N. (2021). Multiplexed live-cell profiling with Raman probes. Nat. Commun..

[61] Li M., Nawa Y., Ishida S. (2022). Label-free chemical imaging of cytochrome P450 activity by Raman microscopy. Commun. Biol..

[62] Hsu C.C., Xu J., Brinkhof B. (2020). A single-cell Raman-based platform to identify developmental stages of human pluripotent stem cell-derived neurons. Proc. Natl. Acad. Sci. U. S. A..

[63] Wang X., Ren L., Diao Z. (2023). Robust spontaneous Raman flow cytometry for single-cell metabolic phenome profiling via pDEP-DLD-RFC. Adv. Sci..

[64] Wang H., Ruan Y., Zhu L. (2021). An integrated electrochemical nanodevice for intracellular RNA collection and detection in single living cell. Angew. Chem. Int. Ed..

[65] Xu Y., Ruan Y., Wang H. (2021). A practical electrochemical nanotool for facile quantification of amino acids in single cell. Small.

[66] Vaneev A.N., Gorelkin P.V., Garanina A.S. (2020). *In vitro* and *in vivo* electrochemical measurement of reactive oxygen species after treatment with anticancer drugs. Anal. Chem..

[67] Zhou L., Kasai N., Nakajima H. (2021). *In situ* single-cell stimulation and real-time electrochemical detection of lactate response using a microfluidic probe. Anal. Chem..

[68] Macosko E.Z., Basu A., Satija R. (2015). Highly parallel genome-wide expression profiling of individual cells using nanoliter droplets. Cell.

[69] Tian L., Jabbari J.S., Thijssen R. (2021). Comprehensive characterization of single-cell full-length isoforms in human and mouse with long-read sequencing. Genome Biol..

[70] Salmen F., De Jonghe J., Kaminski T.S. (2022). High-throughput total RNA sequencing in single cells using VASA-seq. Nat. Biotechnol..

[71] Qiu Q., Hu P., Qiu X. (2020). Massively parallel and time-resolved RNA sequencing in single cells with scNT-seq. Nat. Meth..

[72] Chen W., Guillaume-Gentil O., Rainer P.Y. (2022). Live-seq enables temporal transcriptomic recording of single cells. Nature.

[73] Meyer M., Paquet A., Arguel M.J. (2020). Profiling the non-genetic origins of cancer drug resistance with a single-cell functional genomics approach using predictive cell dynamics. Cell Syst..

[74] Tang Q., Liu L., Guo Y. (2022). Optical cell tagging for spatially resolved single-cell RNA sequencing. Angew. Chem..

[75] Vahid M.R., Brown E.L., Steen C.B. (2023). High-resolution alignment of single-cell and spatial transcriptomes with CytoSPACE. Nat. Biotechnol..

[76] Pan X., Yao H., Zhang S. (2022). Recent progress in mass spectrometry for single-cell metabolomics. Curr. Opin. Chem. Biol..

[77] Men X., Wu C., Zhang X. (2022). Tracking cellular transformation of As(III) in HepG2 cells by single-cell focusing/capillary electrophoresis coupled to ICP-MS. Anal. Chim. Acta.

[78] Lim S.Y., Low Z.E., Tan R.P.W. (2022). Single-cell and bulk ICP-MS investigation of accumulation patterns of Pt-based metallodrugs in cisplatin-sensitive and-resistant cell models. Metallomics.

[79] Meng Y., Cheng X., Wang T. (2020). Micro-lensed fiber laser desorption mass spectrometry imaging reveals subcellular distribution of drugs within single cells. Angew. Chem. Int. Ed..

[80] Cheng X., Yin Z., Rong L. (2020). Subcellular chemical imaging of structurally similar acridine drugs by near-field laser desorption/laser postionization mass spectrometry. Nano Res..

[81] Jia F., Wang J., Zhao Y. (2020). *In situ* visualization of proteins in single cells by time-of-flight–secondary ion mass spectrometry coupled with genetically encoded chemical tags. Anal. Chem..

[82] Végvári Á., Rodriguez J.E., Zubarev R.A. (2022). Single-cell chemical proteomics (SCCP) interrogates the timing and heterogeneity of cancer cell commitment to death. Anal. Chem..

[83] Brunner A.D., Thielert M., Vasilopoulou C. (2022). Ultra-high sensitivity mass spectrometry quantifies single-cell proteome changes upon perturbation. Mol. Syst. Biol..

[84] Zhu G., Zhang W., Zhao Y. (2023). Single-cell metabolomics-based strategy for studying the mechanisms of drug action. Anal. Chem..

[85] Hiyama E., Ali A., Amer S. (2015). Direct lipido-metabolomics of single floating cells for analysis of circulating tumor cells by live single-cell mass spectrometry. Anal. Sci..

[86] Ali A., Abouleila Y., Amer S. (2016). Quantitative live single-cell mass spectrometry with spatial evaluation by three-dimensional holographic and tomographic laser microscopy. Anal. Sci..

[87] Cuypers E., Claes B.S.R., Biemans R. (2022). ‘on the spot’ digital pathology of breast cancer based on single-cell mass spectrometry imaging. Anal. Chem..

[88] Xu S., Liu M., Bai Y. (2021). Multi-dimensional organic mass cytometry: Simultaneous analysis of proteins and metabolites on single cells. Angew. Chem. Int. Ed..

[89] Takeshima H., Yoda Y., Wakabayashi M. (2020). Low-dose DNA demethylating therapy induces reprogramming of diverse cancer-related pathways at the single-cell level. Clin. Epigenet..

[90] Park S.R., Namkoong S., Friesen L. (2020). Single-cell transcriptome analysis of colon cancer cell response to 5-fluorouracil-induced DNA damage. Cell Rep..

[91] Onoda N., Kawabata A., Hasegawa K. (2022). Spatial and single-cell transcriptome analysis reveals changes in gene expression in response to drug perturbation in rat kidney. DNA Res..

[92] Jones-Tabah J., Martin R.D., Tanny J.C. (2021). High-content single-cell Förster resonance energy transfer imaging of cultured striatal neurons reveals novel cross-talk in the regulation of nuclear signaling by protein kinase A and extracellular signal-regulated kinase 1/2. Mol. Pharmacol..

[93] Li H., Gao Y., Xie L. (2021). Prednisone reprograms the transcriptional immune cell landscape in CNS autoimmune disease. Front. Immunol..

[94] Meinsohn M.C., Saatcioglu H.D., Wei L. (2021). Single-cell sequencing reveals suppressive transcriptional programs regulated by MIS/AMH in neonatal ovaries. Proc. Natl. Acad. Sci. U. S. A..

[95] Färkkilä A., Gulhan D.C., Casado J. (2020). Author Correction: Immunogenomic profiling determines responses to combined PARP and PD-1 inhibition in ovarian cancer. Nat. Commun..

[96] Zhong R., Zhang Y., Chen D. (2021). Single-cell RNA sequencing reveals cellular and molecular immune profile in a Pembrolizumab-responsive PD-L1-negative lung cancer patient. Cancer Immunol. Immunother..

[97] Sahu N., Grandi F.C., Bhutani N. (2022). A single-cell mass cytometry platform to map the effects of preclinical drugs on cartilage homeostasis. JCI Insight.

[98] Kashima Y., Shibahara D., Suzuki A. (2021). Single-cell analyses reveal diverse mechanisms of resistance to EGFR tyrosine kinase inhibitors in lung cancer. Cancer Res..

[99] Taavitsainen S., Engedal N., Cao S. (2021). Single-cell ATAC and RNA sequencing reveal pre-existing and persistent cells associated with prostate cancer relapse. Nat. Commun..

[100] Cohen Y.C., Zada M., Wang S. (2021). Identification of resistance pathways and therapeutic targets in relapsed multiple myeloma patients through single-cell sequencing. Nat. Med..

[101] Goveia J., Rohlenova K., Taverna F. (2020). An integrated gene expression landscape profiling approach to identify lung tumor endothelial cell heterogeneity and angiogenic candidates. Cancer Cell.

[102] Wu J., Xiao Y., Sun J. (2020). A single-cell survey of cellular hierarchy in acute myeloid leukemia. J. Hematol. Oncol..

[103] Zhao H., Gao Y., Miao J. (2021). Single-cell RNA-seq highlights a specific carcinoembryonic cluster in ovarian cancer. Cell Death Dis..

[104] Taverna J.A., Hung C.N., Dearmond D.T. (2020). Single-cell proteomic profiling identifies combined AXL and JAK1 inhibition as a novel therapeutic strategy for lung cancer. Cancer Res..

[105] Aggen D.H., Ager C.R., Obradovic A.Z. (2021). Blocking IL1 beta promotes tumor regression and remodeling of the myeloid compartment in a renal cell carcinoma model: Multidimensional analyses. Clin. Cancer Res..

[106] Sung J.Y., Cheong J.H. (2022). Single cell analysis reveals reciprocal tumor-macrophage intercellular communications related with metabolic reprogramming in stem-like gastric cancer. Cells.

[107] Selvin T., Fasterius E., Jarvius M. (2022). Single-cell transcriptional pharmacodynamics of trifluridine in a tumor-immune model. Sci. Rep..

[108] Lee P.J., Ho C.C., Ho H. (2021). Tumor microenvironment-based screening repurposes drugs targeting cancer stem cells and cancer-associated fibroblasts. Theranostics.

[109] Messner C.J., Babrak L., Titolo G. (2021). Single cell gene expression analysis in a 3D microtissue liver model reveals cell type-specific responses to pro-fibrotic TGF-β1 stimulation. Int. J. Mol. Sci..

[110] Smits L.M., Magni S., Kinugawa K. (2020). Single-cell transcriptomics reveals multiple neuronal cell types in human midbrain-specific organoids. Cell Tissue Res..

[111] Zhao Y., Li Z., Zhu Y. (2021). Single-cell transcriptome analysis uncovers intratumoral heterogeneity and underlying mechanisms for drug resistance in hepatobiliary tumor organoids. Adv. Sci..

[112] Inak G., Rybak-Wolf A., Lisowski P. (2021). Defective metabolic programming impairs early neuronal morphogenesis in neural cultures and an organoid model of Leigh syndrome. Nat. Commun..

[113] Yuan Z., Fan X., Zhu J.J. (2021). Presence of complete murine viral genome sequences in patient-derived xenografts. Nat. Commun..

[114] Ediriwickrema A., Aleshin A., Reiter J.G. (2020). Single-cell mutational profiling enhances the clinical evaluation of AML MRD. Blood Adv..

[115] Rozenblatt-Rosen O., Regev A., Oberdoerffer P. (2020). The human tumor atlas network: Charting tumor transitions across space and time at single-cell resolution. Cell.

[116] Li M., Zhang X., Ang K.S. (2022). DISCO: A database of Deeply Integrated human Single-Cell Omics data. Nucleic Acids Res..

[117] Van de Sande B., Lee J.S., Mutasa-Gottgens E. (2023). Applications of single-cell RNA sequencing in drug discovery and development. Nat. Rev. Drug Discov..

[118] Cao Y., Su B., Guo X. (2020). Potent neutralizing antibodies against SARS-CoV-2 identified by high-throughput single-cell sequencing of convalescent patients’ B cells. Cell.

[119] Qi R., Zou Q. (2023). Trends and potential of machine learning and deep learning in drug study at single-cell level. Research.

